# Catechol‐Containing Poly(2‐isopropyl‐2‐oxazoline): Synthesis and Thermoresponsive Behavior in Aqueous Salt Solutions

**DOI:** 10.1002/marc.202500719

**Published:** 2025-10-30

**Authors:** Niclas Madaj, Nils Lüdecke, Sergio Kogikoski, Ilko Bald, Helmut Schlaad

**Affiliations:** ^1^ Institute of Chemistry University of Potsdam Potsdam Germany

**Keywords:** catechol, copolymerization, lower critical solution temperature (LCST), poly(2‐oxazoline), post‐polymerization modification, Raman spectroscopy

## Abstract

Catechol‐containing poly(2‐isopropyl‐2‐oxazoline)s (PiPOx) were obtained either by functionalization of partially hydrolyzed PiPOx with 3,4‐dimethoxybenzoyl chloride or by microwave‐assisted cationic ring‐opening copolymerization of 2‐isopropyl‐2‐oxazoline with 2‐(3,4‐dimethoxyphenyl)‐2‐oxazoline, followed by quantitative deprotection of methylated catechols. All synthesized polymers were soluble in aqueous salt solutions at room temperature and showed thermoresponsive LCST behavior. The cloud point temperature (*T*_CP_) remained virtually unchanged in the presence of different cations, while it was strongly affected by chaotropic and kosmotropic anions, which is in line with the Hofmeister series. Intriguingly, *T*_CP_ was lower for the catechol‐containing PiPOx as compared to the protected precursor copolymer and PiPOx, which is rationalized by hydrogen bonding interactions between catechol and tertiary amide units of the polymer backbone, as indicated by Raman spectroscopy, decreasing the hydration level of the polymer.

## Graphical abstract

Polymers carrying catechol groups are interesting mussel‐inspired materials for wet adhesion applications or metal ion complexation for wastewater treatment. Here, catechol‐containing poly(2‐isopropyl‐2‐oxazoline)s were synthesized via microwave‐assisted cationic (co)polymerization and examined with regard to their thermoresponsive LCST behavior in aqueous salt solutions.

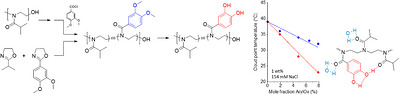

## 1 Introduction

Polymers containing catechol units are an interesting class of materials with potentially superior chelating and adhesive properties [1]. Mussels are the most prominent example of marine organisms that make use of catechol‐containing proteins to attach to many different kinds of surfaces in wet marine surroundings [2] through coordination, hydrogen bonding, π‐π‐stacking, covalent bonding, or electrostatic attraction [3, 4, 5]. However, protein attachment to surfaces is enhanced by positively charged residues located within the same molecule [6, 7, 8, 9].

Following this bioinspired design principle, we earlier reported on the synthesis of hydrophilic cationic catechol‐containing copolymers based on poly(2‐ethyl‐2‐oxazoline) [10] and their use for the effective coating of different substrates, including gold, iron, glass, and polystyrene surfaces [11]. Choosing poly(2‐oxaoline)s as a pseudopeptide or peptide‐like polymer platform [12, 13] was for three main reasons, which are the convenient availability of well‐defined materials by microwave‐assisted living cationic ring‐opening polymerization (CROP) [14], the possibility of introducing diverse polymer functionalities [15, 16, 17, 18, 19, 20, 21, 22, 23, 24] including catechols [25, 26, 27], and the easiness of generating positively charged residues by simple acidic hydrolysis [28].

Motivated by the promising surface coating performance of the catechol‐containing copoly(2‐ethyl‐2‐oxazoline)s, we wanted to extend the toolbox of bioinspired adhesive copolymers by copoly(2‐isopropyl‐2‐oxazoline)s. Poly(2‐isopropyl‐2‐oxazoline) (PiPOx) exhibits thermoresponsive LCST (= lower critical solution temperature) behavior in aqueous solution with a cloud point temperature of around 36°C [16, 17, 29], and it can be crystallized in hot water and precipitated as crystalline nanofibers or porous microspheres [30, 31, 32]. Hence, catechol‐containing copoly(2‐isopropyl‐2‐oxazoline)s would offer the possibility to prepare thermoresponsive polymer coatings on metal surfaces or particles and might also be effective for the complexation and precipitation of metal ions from aqueous solution (water purification).

Here, we want to present the optimized synthesis protocol for catechol‐containing copoly(2‐isopropyl‐2‐oxazoline)s by either functionalization of partially hydrolyzed PiPOx or statistical copolymerization of 2‐isopropyl‐2‐oxazoline and 2‐(3,4‐dimethoxyphenyl‐2‐oxazoline) and subsequent quantitative deprotection (also solving the earlier observed issue of incomplete demethylation of protected catechol units in copoly(2‐ethyl‐2‐oxazoline)s [10]). Copolymers were characterized by NMR and Raman spectroscopy and size exclusion chromatography (SEC). The copolymers containing 1.9‐7.9 mol% catechol units (no positively charged residues) were examined with respect to their LCST behavior in different aqueous salt solutions, by turbidimetric measurements, especially focusing on the effect of catechol units on polymer solubility and cloud point temperature and salting‐in and out effects (Hofmeister series).

## 2 Experimental Section

### 2.1 Chemicals

Chemicals were purchased from commercial sources and used as received, unless otherwise stated. Methyl tosylate (MeOTos) (98%), 3,4‐dimethoxybenzoyl chloride (DMBzCl) (98%), triethylamine (≥ 99.5%), pyridine (≥ 99%), boron tribromide (100%, 1 m in anhydrous dichloromethane), triethylsilane (99%), calcium chloride hexahydrate (98%), and guanidinium chloride (GdmCl) (100%): Sigma‐Aldrich. Isobutyronitrile (99%), tris(pentafluorophenyl)borane (97%), and magnesium sulfate (99.5%, anhydrous): Alfa Aesar. Ethanolamine (99%), sodium hydroxide (98.5%), *N*,*N*’‐diisopropylcarbodiimide (99%), sodium perchlorate (> 99%), and zinc chloride (99.99%): Acros Organics. Cadmium(II) acetate (99.999%), cadmium(II) acetate dihydrate (≥ 99%), and iron(III) chloride (≥ 98.5%): Carl Roth. 3,4‐Dimethoxybenzonitrile (98%), calcium hydride (CaH_2_) (**∼** 93%, 0 to 2 mm grain size), aluminum oxide (AlO_x_) (100%, neutral, Brockmann I, 60 Å, 40 to 300 µm), chlorobenzene (99.5%, extra dry over molecular sieve), and chloroform‐d (CDCl_3_) (99.8% atom D): Thermo Scientific. Hydrochloric acid (37%), sodium chloride (≥ 99.5%), and sodium sulfate (99.7%): Fisher Chemical. 1,2‐Dihydroxybenzene (>98.0%): Fluka. Potassium hydroxide (85.0 to 100.5%): neoLab Migge Laborbedarf‐Vertrieb GmbH. Aluminum iodide (AlI_3_) (95%): ABCR. Silica gel 60 (neutral, 40 to 63 µm): Avantor. Acetonitrile (≥ 99.9%, hypergrade for LC‐MS): Merck. Ethyl acetate (99.8%) and methanol (technical grade): VWR. Dichloromethane (DCM) (technical grade): Stockmeyer Chemie. Dimethyl sulfoxide‐d_6_ (DMSO‐d_6_) (99.8% atom D) and deuterium oxide‐d_2_ (D_2_O) (99.9% atom D): Deutero GmbH. Methyl tosylate, acetonitrile, and DCM were dried over CaH_2_ and distilled prior to use. DCM used for polymer purification was distilled in a rotary evaporator.

### 2.2 Monomers

#### 2.2.1 2‐Isopropyl‐2‐Oxazoline (iPOx)

A dried 100 mL double‐neck round‐bottom flask with N_2_‐atmosphere was equipped with isobutyronitrile (38.50 g, 0.55 mol, 50.00 mL), and Cd(II) acetate (6.36 g, 0.028 mol) under inert conditions. The mixture was heated to 130°C under reflux conditions and stirred for 5 min. Subsequently, ethanolamine (35.73 g, 0.58 mol, 35.03 mL) was added dropwise within 20 min. This mixture was then stirred for another 24 h at 130°C under reflux conditions. After cooling to ambient temperature, the crude product was isolated from the reaction soil by vacuum distillation (stepwise from 300 to 100 mbar, and from 100°C to 115°C). iPOx fractions with residual isobutyronitrile were combined and mixed with CaH_2_ to dry. The product was obtained after vacuum distillation from CaH_2_ (200 to 150 mbar and ambient temperature for isobutyronitrile; 100 mbar and 75°C for iPOx) as a colorless liquid; yield: 45.01 g (0.40 mol, 72%).

#### 2.2.2 2‐(3,4‐Dimethoxyphenyl)‐2‐oxazoline (DMPOx)

A dried 100 mL three‐neck round‐bottom flask with N_2_‐atmosphere was equipped with 3,4‐dimethoxybenzonitrile (8.33 g, 0.05 mol), Cd(II) acetate dihydrate (0.54 g, 0.002 mol), and chlorobenzene (30 mL) under inert conditions. The mixture was heated to 140°C under reflux conditions and stirred for 5 min. Heated (activated) molecular sieve (3 Å) was added. Subsequently, ethanolamine (4.10 g, 0.067 mol, 4.02 mL) was added dropwise with a syringe within 5 min. This mixture was then stirred for another 24 h at 140°C under reflux conditions. After cooling to ambient temperature, the reaction was quenched with deionized water (40 mL). The two phases were separated, and the aqueous phase was extracted with ethyl acetate (3 × 100 mL). The combined organic layers were dried over magnesium sulfate (30 min), filtered, and volatiles were removed in vacuum. The crude product was purified by column chromatography (silica gel, ethyl acetate, R_f_ 0.26) and isolated as an off‐white solid; yield: 8.78 g (0.04 mol, 85%).

### 2.3 Polymerizations and Post‐Polymerization Modifications

Each polymerization and kinetic experiment corresponds to its own reaction and needs to be done in a separate microwave vial, placed in the microwave reactor (CEM Discover) one after the other, heated and stirred for a predetermined reaction time, quenched, and analyzed separately.

#### 2.3.1 Homopolymerization of iPOx

A dry microwave vial was filled with 3.0 mL of a 2.0 m stock solution of iPOx in acetonitrile and 0.3 mL of a 0.2 m stock solution of MeOTos in acetonitrile under N_2_ atmosphere. The vial was sealed with a microwave septum under N_2_‐atmosphere, placed in the microwave reactor, and the pressure sensor was placed over the septum. The mixture was heated to 140°C (power: 200 W) and stirred for 15 min. After cooling quickly to 50°C with an air pressure flow, the reaction mixture was terminated with 2 m KOH solution in MeOH/H_2_O 1:1 (v/v) (5 drops, excess) and stirred for 1 h at ambient temperature. Solvents were removed in vacuum, and the residue was redissolved in MeOH/DCM 1:1 (v/v) (2 mL) and purified by passing through a short aluminum oxide column. The dried PiPOx was dissolved in deionized water (**∼**2 mL), dialyzed (regenerated cellulose, MWCO 1 kDa) against ultrapure water (**∼**1 L), and freeze‐dried; yield: 475 mg (70%), colorless solid.

#### 2.3.2 Copolymerization of iPOx and DMPOx

Binary iPOx/DMPOx monomer solutions were prepared from 2.0 m stock solutions of iPOx and DMPOx in acetonitrile under N_2_ atmosphere: 2.94 mL/0.06 mL (2 mol% DMPOx), 2.85 mL/0.15 mL (5 mol% DMPOx), 2.70 mL/0.30 mL (10 mol% DMPOx), and 2.40 mL/0.60 mL (20 mol% DMPOx). To the monomer solutions were added 0.3 mL of a 0.2 M solution of MeOTos in acetonitrile under N_2_ atmosphere. The vials were sealed with a microwave septum, placed in the microwave reactor, and the pressure sensor was placed over the septum. The mixtures were heated to 140°C (power: 200 W) and stirred for 30 min. After cooling quickly to 50°C with an air pressure flow, the crude reaction mixtures were terminated with 2 m KOH solution in MeOH/H_2_O 1:1 (v/v) (5 drops, excess) and stirred for 1 h at ambient temperature. Solvents were removed in vacuum, and the residues were redissolved in MeOH/DCM 1:1 (v/v) (2 mL) and purified by passing through a short aluminum oxide column. The dried copolymers were dissolved in deionized water (**∼**2 mL), dialyzed (regenerated cellulose, MWCO 1 kDa) against ultrapure water (**∼**1 L), and freeze‐dried; yield: ∼70%, colorless solids.

#### 2.3.3 Kinetic Measurements

iPOx and binary iPOx/DMPOx monomer solutions were prepared from 2.0 m stock solutions of iPOx and DMPOx in acetonitrile under N_2_ atmosphere: 3.00 mL/0 mL (iPOx), 2.94 mL/0.06 mL (2 mol% DMPOx), 2.47 mL/0.13 mL (5 mol% DMPOx), 3.15 mL/0.35 mL (10 mol% DMPOx), 2.80 mL/0.70 mL (20 mol% DMPOx), and 0.35 mL/3.15 mL (90 mol% DMPOx). 0.5 mL of the monomer solutions was transferred to dry microwave vials and mixed with 0.05 mL of a 0.2 m solution of MeOTos in acetonitrile under N_2_ atmosphere. Every vial was sealed with a microwave septum, placed in the microwave reactor, and the pressure sensor was placed over the septum. The mixture was heated to 140°C (power: 200 W) and stirred for a predetermined reaction time (2–15 min). After cooling quickly to 50°C with an air pressure flow, 4 drops of the crude reaction mixture were combined with wet DMSO‐d_6_ (0.60 mL) and analyzed by ^1^H NMR spectroscopy.

#### 2.3.4 Partial Acidic Hydrolysis

A microwave vial was equipped with PiPOx (200 mg) and 6 m aqueous HCl solution (4 mL). The vial was sealed with a microwave septum, placed in the microwave reactor, and the pressure sensor was placed over the septum. The mixture was heated to 100°C (power: 200 W) and stirred for a predetermined reaction time (5–25 min). After cooling quickly to 50°C with an air pressure flow, the crude reaction mixture was neutralized with 6 m aqueous NaOH. The polymer was purified by dialysis (regenerated cellulose, MWCO 1 kDa) against ultrapure water (2 × **∼**1 L) and freeze‐dried; yield: 70% to 90%, colorless solids.

#### 2.3.5 Functionalization of Partially Hydrolyzed PiPOx

A dried 10 mL round‐bottom flask was equipped with partially hydrolyzed PiPOx (100 mg, **∼**0.06 mmol EI units) and dry DCM (2 mL) under N_2_ atmosphere. The mixture was cooled to 0°C and stirred for 5 min. Then, 3,4‐dimethoxybenzoyl chloride (24.57 mg, 0.122 mmol, 2 equiv. with respect to EI units) was added. Subsequently, either triethylamine (0.02 mL) or pyridine (0.01 mL) was added dropwise with a syringe within a few seconds, and the mixture was stirred for another 3 h or 4.5 h at ambient temperature. Afterward, volatiles were removed in vacuum, and the crude products were redissolved in ultrapure water (as little as possible). The cloudy solutions were left to stand until the residues had settled. The supernatant solutions were separated and dialyzed (regenerated cellulose, MWCO 1 kDa) against ultrapure water (2 × **∼**1 L). Yield: 84.9 mg and 77.0 mg, respectively, colorless solids.

#### 2.3.6 Demethylation of 3,4‐Dimethoxyphenyl Units

A dried 250 mL round flask was equipped with a protected catechol‐containing copolymer (250 mg, **∼**0.02 mmol for a polymer with 10 mol% DMPOx and DP 100) and dry DCM (65 mL). The mixture was cooled to 0°C and stirred for 5 min. Subsequently, 1 m boron tribromide solution in DCM (9.79 mL, 4 equiv. BBr_3_ with respect to amide (DP 100)) was added dropwise with a syringe within 2 min. The mixture was stirred for 18 h at ambient temperature. The reaction was quenched by the addition of MeOH (75 mL, excess), stirred for 5 min, and volatiles were removed in vacuum. The crude polymer was dissolved in ultrapure water, dialyzed (regenerated cellulose, MWCO 1 kDa) against ultrapure water (2 × **∼**1 L), and freeze‐dried; yield: ≥ 90%, colorless solid.

### 2.4 Analytical Instrumentation and Methods

Nuclear magnetic resonance (NMR) spectra were recorded on a Bruker Avance NEO 400 MHz spectrometer. CDCl_3_, DMSO‐*d_6_*, or D_2_O were used as deuterated solvents. Signals were referenced to the solvent residual peaks *δ* 7.26 ppm, 2.50 ppm, or 4.79 ppm, respectively. Size exclusion chromatography (SEC) with simultaneous UV (*λ* = 285 nm) and RI (differential refractive index) detection was performed with *N*‐methyl‐2‐pyrrolidone (NMP) + 0.5 wt% LiBr as the eluent (flow rate: 0.5 mL·min^−1^) at ambient temperature. The stationary phase was a 300 **×** 8 mm^2^ PSS‐GRAM analytical linear column (particle size 7 µm, separation range 10^2^–10^6^ Da). Solutions containing ∼0.1 wt% polymer were filtered through 0.45 µm syringe filters; the injected volume was 100 µL. Polystyrene standards (PSS, Mainz, Germany) were used for calibration. UV–vis spectra were recorded with a Perkin Elmer Lambda 2 spectrometer at room temperature. Turbidity curves were recorded with a turbidimetric photometer TP1 (Elmar Tepper, Germany), operating at a laser wavelength of *λ* 670 nm, at a heating/cooling rate of 1 K min^−1^. Aqueous solutions containing 1.0 wt% polymer with or without different salt concentrations were filtered through 0.45 µm syringe filters. The temperature at which the transmission was reduced to 80% in the heating cycle was taken as the cloud point temperature (*T*_CP_). Raman spectroscopy measurements were performed on a LabRam Evolution (Horiba JobinYvon, France) spectrometer. The spectra were collected using a 532 nm laser, with about 1 mW of power at the focal point. A 50X (N.A. 0.5) objective was used to focus on the surface. Samples were measured in a completely wet as well as a dry state. Briefly, about 5 mg of polymer was placed on a silicon substrate, and 20 µL of water was added, generating a highly concentrated polymer dispersion. After this, the laser was focused closer to the center of the drop, and the spectra were collected continuously for 30 min (10 s of accumulation for each spectrum). The obtained data were baseline corrected using the LabVIEW 6 software of the equipment.

## 3 Results and Discussion

### 3.1 Synthesis of Catechol‐Functionalized Copoly(2‐Isopropyl‐2‐Oxazoline)

**SCHEME 1:**
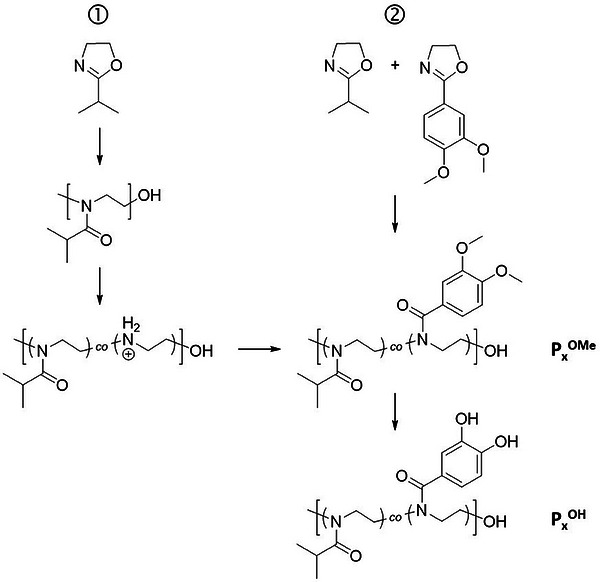
Two alternate reaction pathways toward catechol‐containing copoly(2‐isopropyl‐2‐oxazoline). Sample code: subscript x denotes the mole fraction (%) of 3,4‐dimethoxyphenyl units in the copolymer, and the superscript OMe/OH indicates whether the catechol hydroxyl groups are protected as methyl ethers or free, respectively.

We explored two different pathways toward 3,4‐dimethoxyphenyl‐containing poly(2‐isopropyl‐2‐oxazoline) (PiPOx), either by (1) functionalization of partially hydrolyzed PiPOx with 3,4‐dimethoxybenzoyl chloride or by (2) copolymerization of 2‐isopropyl‐2‐oxazoline (iPOx) with 2‐(3,4‐dimethoxyphenyl)‐2‐oxazoline (DMPOx), which, after deprotection, i.e., demethylation of the 3,4‐dimethoxyphenyl units, give the catechol‐containing copolymer (see Scheme 1).

#### 3.1.1 Microwave‐Assisted Synthesis of Poly(2‐Isopropyl‐2‐Oxazoline), Partial Acidic Hydrolysis, and Post‐Polymerization Modification

First, we performed the cationic ring‐opening polymerization (CROP) of iPOx, initiated by methyl tosylate (MeOTos), in acetonitrile solution at 140°C in a microwave reactor ([iPOx]_0_ = 1.82 M and [iPOx]_0_/[MeOTos]_0_ = 100, corresponding to a targeted number‐average molar mass (*M*_n_) of 11.3 kg mol^−1^ at full monomer conversion). Since the polymerization of iPOx was expected to be slower than that of 2‐ethyl‐2‐oxazoline (EtOx) [10, 27], the polymerization was performed at a higher concentration of reactants, and the power of the microwave reactor was increased from 100 W to 200 W. A series of polymerizations was quenched after predetermined reaction times (3, 6, 9, 12, and 15 min), and crude reaction mixtures were analyzed by ^1^H NMR spectroscopy for the determination of monomer conversions (see Figure S1, Supporting Information). The polymerization was found to follow a pseudo‐first‐order kinetics with an apparent rate constant of propagation of *k*_app_ = 0.29 min^−1^, which translates into a rate constant of propagation of *k*_p_ = *k*_app_/[MeOTos]_0_ = 0.27 L mol^−1^ s^−1^, and quantitative monomer conversion was achieved within 15 min (Figure S2, Supporting Information). Notably, however, the polymerization of iPOx was faster than what was reported earlier by Hoogenboom et al. (*k*_p_ = 0.048 L mol^−1^ s^−1^; [iPOx]_0_ = 4 m, [iPOx]_0_/[MeOTos]_0_ = 50, acetonitrile, microwave, 140°C) [33] and much faster than that with conventional heating in an oil bath at 70°C, for which we achieved quantitative monomer conversion only after 7 days (highlighting the benefit of microwave heating under autoclave conditions to drastically shorten reaction times). The polymerization was repeated several times for accumulation of sufficient amounts of PiPOx (a single experiment in the microwave reactor produces about 450 mg of material, after purification), which typically exhibited an apparent number‐average molar mass (*M*_n_^app^) of ∼10 kg mol^−1^, as determined by SEC with polystyrene calibration, and a low dispersity index (*Ð*) of ∼1.13 (Figure S2, Supporting Information).

**FIGURE 1:**
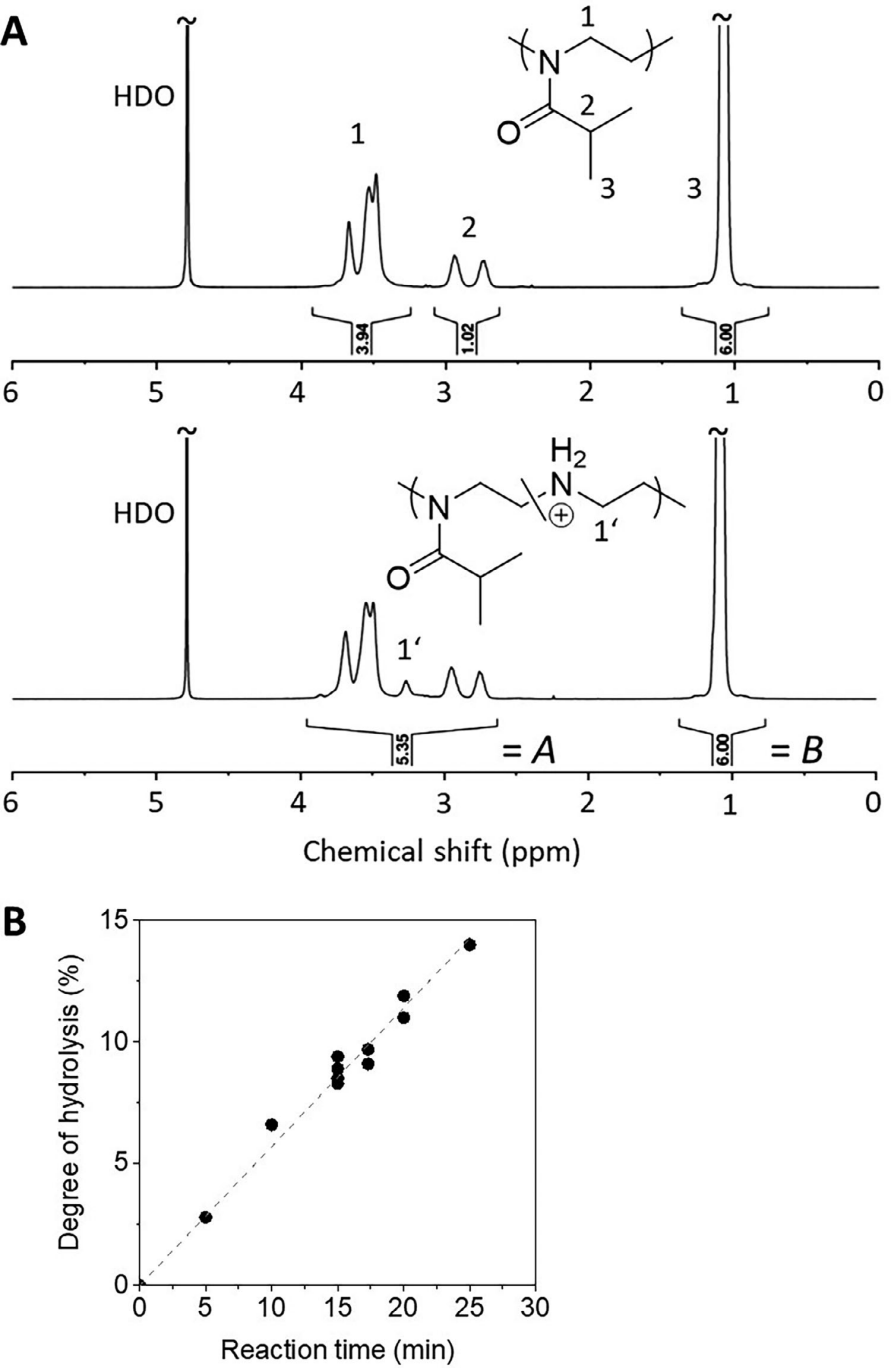
Microwave‐assisted acidic hydrolysis of PiPOx in 6 m HCl solution at 100°C: (A) Exemplary ^1^H NMR (400 MHz, D_2_O) spectra of the original PiPOx and partially hydrolyzed PiPOx obtained after a reaction time of 15 min; (B) Plot of the degree of hydrolysis as a function of reaction time.

Afterward, the PiPOx (5 wt%) was subjected to microwave‐assisted acidic hydrolysis in 6 m hydrochloric acid at 100°C [34], aiming at partially hydrolyzed PiPOx samples containing about 5–20% ethylene imine (EI) units. Notably, the hydrolysis of PiPOx requires harsher conditions than that of, for instance, PEtOx [35] and was not effective in a less concentrated 1 m aqueous HCl solution, possibly also as a matter of the insolubility of PiPOx at elevated temperature. Partially hydrolyzed PiPOx samples were produced for different reaction times (5–25 min) and were analyzed by ^1^H NMR spectroscopy (Figure 1A; Figure S3, Supporting Information). The presence of protonated EI units could be confirmed by a broad proton signal at *δ* ∼3.1–3.3 ppm, which, however, was not always very pronounced and clearly separated from the methylene and methine signals of PiPOx, thus impeding the accurate analysis of the degree of hydrolysis (*x*). We therefore used the peak integrals *A* at *δ* 2.6–3.9 ppm (CH_2_, CH, PiPOx, (1‐*x*) 5H and CH_2_, EI, *x* 4H) and *B* at *δ* 1.1 ppm (CH_3_, PiPOx, (1‐*x*) 6H) and calculated the degree of hydrolysis by *x* = (1+4/(6*A*/*B*−5))^−1^ (see Supporting Information). Based on this analysis, the degree of hydrolysis of PiPOx was found to correlate linearly with reaction time (Figure 1B), thus allowing for estimating the amount of EI units in the copolymer by *x* (%) = 0.57 min^−1^ × time (min) (at least for a degree of hydrolysis of up to about 15%).

Notably, yet to be confirmed, the partial acidic hydrolysis using a high excess of acid over amide groups should ideally result in the formation of evenly distributed isolated EI units along the polymer chain. However, it has been reported that the protonated amine may activate a neighboring amide group and accelerate its hydrolysis [28], which would eventually produce dimeric or even higher EI block sequences.

**FIGURE 2:**
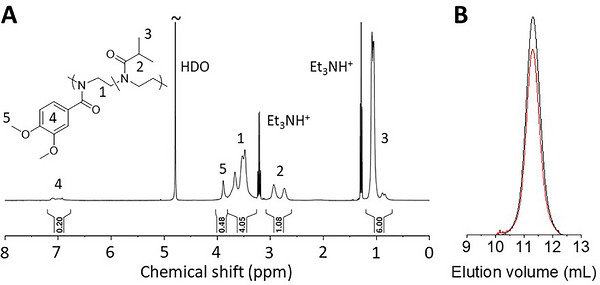
(A) ^1^H NMR (400 MHz, D_2_O) spectrum and (B) SEC‐RI/UV_285 nm_ traces (eluent: NMP; RI black line, UV red line) of copolymer **P_6.5_^OMe^** obtained by functionalization of partially hydrolyzed PiPOx with DMBzCl/triethylamine.

Exemplarily, we used a partially hydrolyzed PiPOx with a degree of hydrolysis of 6.5% for the functionalization with 3,4‐dimethoxybenzoyl chloride (DMBzCl). The reaction was performed in DCM solution (∼50 mg mL^−1^ polymer, [DMBzCl]_0_/[EI]_0_ = 2) for 3 h at room temperature in the presence of triethylamine as a base. ^1^H NMR analysis (Figure 2A) suggested a quantitative degree of functionalization, i.e., ∼6.5 mol% of 3,4‐dimethoxyphenyl units in the copolymer (referred to as **P_6.5_^OMe^**), but also revealed a contamination with triethylammonium chloride (see also [24]). Notably, all efforts to completely remove the salt have failed so far. SEC analysis (Figure 2B) further confirmed the presence of 3,4‐dimethoxyphenyl units in the copolymer by the UV trace at 285 nm (see below) and preservation of the narrow molar mass distribution (*M*_n_^app^ 10.7 kg mol^−1^, *Ð* 1.12).

We also performed the functionalization reaction with DMBzCl using pyridine as a base. According to ^1^H NMR analysis, however, the degree of functionalization just reached about 20% after a reaction time of 4.5 h. Here, the partially functionalized copolymer product did not contain any pyridinium chloride salt impurities (see Figure S4, Supporting Information).

It should be noted that the incomplete functionalization of partially hydrolyzed PiPOx is the preferred route toward cationic catechol‐containing copoly(2‐isopropyl‐2‐oxazoline)s as mussel‐inspired adhesives for efficient coating of metal surfaces [11]. The alternate route of partial acidic hydrolysis of catechol‐containing copoly(2‐isopropyl‐2‐oxazoline) (6 m HCl, 100°C) instead suffers from simultaneous cleavage of isopropyl and aromatic side chains (data not shown).

#### 3.1.2 Microwave‐Assisted Copolymerization of 2‐Isopropyl‐2‐Oxazoline and 2‐(3,4‐Dimethoxyphenyl)‐2‐Oxazoline

**FIGURE 3:**
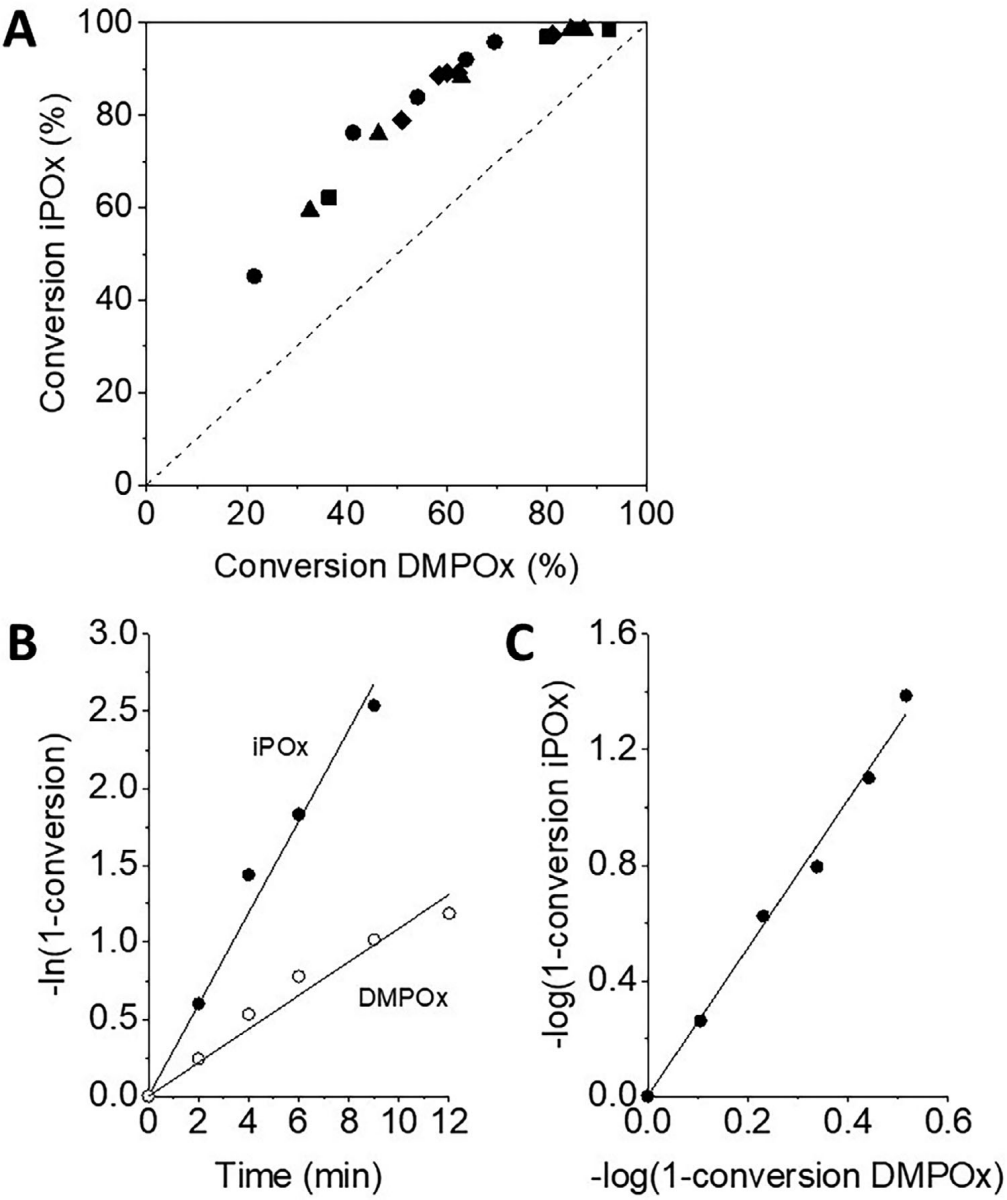
(A) Monomer conversions for the microwave‐assisted copolymerization of mixtures of 2‐isopropyl‐2‐oxazoline (iPOx) and 2‐(3,4‐dimethoxyphenyl)‐2‐oxazoline (DMPOx) 98:2 (circles), 95:5 (squares), 90:10 (triangles), and 80:20 (diamonds) in acetonitrile solution at 140°C. (B) Pseudo first‐order time‐conversion plots for the polymerization of iPOx (closed circles) and DMPOx (open circles) in the 98:2 mixture. (C) Jaacks plot for the copolymerization of the iPOx/DMPOx 98:2 mixture; slope of linear regression line: 2.6 ± 0.1.

The microwave‐assisted statistical copolymerizations of binary monomer mixtures of iPOx and 2‐(3,4‐dimethylphenyl)‐2‐oxazoline (DMPOx) (2, 5, 10, or 20 mol%) were performed in the same way as the homopolymerization of iPOx ([iPOx+DMPOx]_0_/[MeOTos]_0_ = 100). The polymerizations were quenched with wet DMSO‐d_6_ after predetermined reaction time and analyzed by ^1^H NMR spectroscopy for determination of the monomer conversions (considering the triplet signals of oxazoline methylene protons at 4.37 ppm (DMPOx) and 4.15 ppm (iPOx) relative to the acetonitrile solvent peak at 2.08 ppm; spectra are shown in the Figures S5–S8, Supporting Information). The iPOx was always consumed faster than DMPOx (Figure 3A), which is rather expected since 2‐oxazolines with aliphatic substituents are usually more reactive than those with aromatic substituents. The polymerization of both monomers follows pseudo‐first‐order kinetics (indicating the absence of termination reactions) with apparent rate constants of *k*_app_ = (0.30 ± 0.01) min^−1^ (iPOx) and (0.11 ± 0.01) min^−1^ (DMPOx), as revealed from the copolymerization of the iPOx/DMPOx 98:2 mixture (Figure 3B). Evaluation of the reactivity parameters according to Jaacks [36] (which applies for ideal copolymerizations with one of the monomers being in large excess, here: iPOx/DMPOx 98:2, Figure 3C) yields *r*_1_ (iPOx) = *r*_2_^−1^ (DMPOx) = 2.6 ± 0.1. (Notably, kinetic analysis of the copolymerization of a DMPOx/iPOx 90:10 mixture (Figure S9, Supporting Information) yields *r*_2_ (DMPOx) = 0.46 ± 0.01, see Figure S10 (Supporting Information), thus r_1_⋅r_2_ = 1.2 ≈ 1, as required for an ideal copolymerization). Accordingly, since *r*_1_ > *r*_2_, the statistical copolymers exhibit a slight gradient structure, and the DMPOx units are not ideally randomly distributed along the polymer chain. It should be mentioned that the copolymerization of EtOx/DMPOx (90:10) produced copolymers with steeper gradient structure [10], *r*_1_ (EtOx) = 7.0 ± 0.3 (Figure S11, Supporting Information), due to the faster polymerization of EtOx as compared to iPOx.

Like for EtOx/DMPOx [10], the copolymer molar mass (*M*_n_^app^, by SEC) was found to increase linearly with monomer conversion during the copolymerization of iPOx/DMPOx (see Figure S12, Supporting Information), as expected for a living copolymerization process.

**FIGURE 4:**
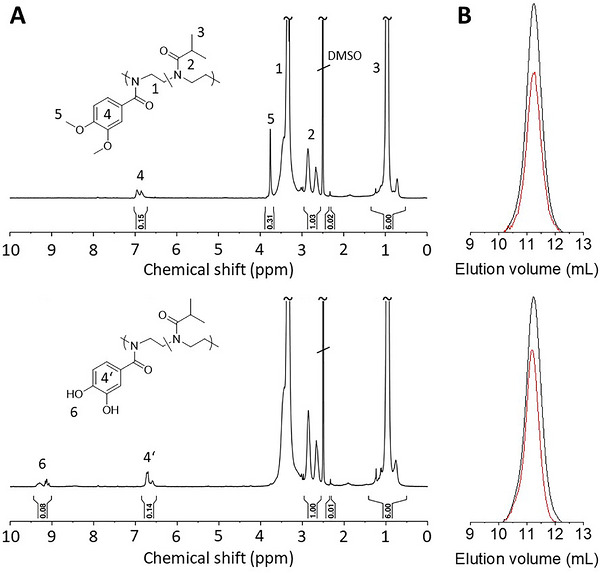
(A) Exemplary ^1^H NMR (400 MHz, DMSO‐d_6_) spectra and (B) SEC‐RI/UV_285 nm_ traces (eluent: NMP; RI black line, UV red line) of copolymer **P_4.8_^OMe^** (top) and corresponding deprotected copolymer **P_4.8_^OH^** (bottom).

Larger amounts of three gradient copolymers were then synthesized by copolymerization of different monomer mixtures (2, 5, and 10 mol% DMPOx, [iPOx+DMPOx]_0_/[MeOTos]_0_ = 100) at 140°C for 30 min. (A copolymer containing 20 mol% DMPOx was discarded because of its poor solubility in water at room temperature, see below.) According to ^1^H NMR analyses, considering the signals at *δ* 6.9 ppm (aryl, 3H) and *δ* 1.0 ppm (CH_3_, isopropyl, 6H) (see Figure 4A, top; Figure S13, Supporting Information), the samples contained ∼1.9 mol% (→ **P_1.9_^OMe^**), 4.8 mol% (→ **P_4.8_^OMe^**), and 7.9 mol% (→ **P_7.9_^OMe^**) of 3,4‐dimethoxyphenyl units. The mole fractions of 3,4‐dimethoxyphenyl units in the copolymers were always found to be somewhat lower than those of DMPOx in the initial monomer feeds, which is due to a less‐than‐quantitative conversion of DMPOx. SEC analyses further confirmed the incorporation of 3,4‐dimethoxyphenyl units in the copolymers by the UV absorption trace at *λ* = 285 nm (DMPOx: *λ*_max_ 288 nm, acetonitrile). According to SEC, the three copolymers exhibited apparent number‐average molar masses in the expected range of 10.2–11.3 kg mol^−1^ and narrow monomodal molar mass distributions with *Ð* 1.13–1.14 (Figure 4B, top; Figure S13, Supporting Information).

#### 3.1.3 Deprotection (Demethylation) of 3,4‐Dimethyoxyphenyl Units

The next step was the deprotection (demethylation) of the 3,4‐dimethoxyaryl units to release the catechol functional groups along the copolymer chain (Scheme 1). In our first attempt, as described earlier [10, 27], we treated the protected copolymers with aluminum triiodide/*N,N’*‐diisopropylcarbodiimide (1.1 equiv. with respect to aryl groups) in acetonitrile suspension at 80°C, however, cleavage of the methyl aryl ethers proceeded to just about 40% or less. We also applied milder protocols, which are the treatment with boron tribromide (∼6 equiv.) in dichloromethane at 0°C followed by stirring overnight at room temperature [37] and reaction with triethylsilane/tris(pentafluorophenyl)borane and subsequent acidic hydrolysis at room temperature [38], but could not achieve conversions >50% (data not shown). We hypothesized that the non‐quantitative cleavage of the methyl aryl ethers was simply due to an insufficient effective amount of Lewis acid as a matter of competitive coordination of the Lewis acid to the amide groups of the copoly(2‐oxazoline) chain. We therefore repeated the deprotection of the copolymers **P_1.9_^OMe^**, **P_4.8_^OMe^**, and **P_7.9_^OMe^** with boron tribromide and used a much larger excess of the Lewis acid of 4 equiv. with respect to amide groups, which then resulted in quantitative cleavage of the methyl aryl ethers within 18 h, as confirmed by ^1^H NMR analyses of the isolated samples **P_1.9_^OH^**, **P_4.8_^OH^**, and **P_7.9_^OH^** in DMSO‐d_6_ (Figure 4A, bottom; Figure S14, Supporting Information). The proton NMR signal of the methoxy aryl ether at *δ* 3.76 ppm was completely absent, and instead the signal of the released catechol OH protons appeared at *δ* 9.1–9.3 ppm. Any cleavage of aromatic units did not seem to happen, as suggested by the consistent peak integral ratio of isopropyl and phenyl protons. SEC analyses revealed virtually no or marginal differences between the protected and deprotected samples (*M*_n_^app^ 10.7–11.3 kg mol^−1^, *Ð* 1.15–1.16) (Figure 4B, bottom; Figure S14, Supporting Information), indicating that the copolymer chains were not degraded during the treatment with boron tribromide. Notably, Raman spectroscopic analysis of sample **P_7.9_^OH^** did not reveal the presence of oxidized catechols, i.e. quinones (see below).

### 3.2 Thermoresponsive Behavior in Aqueous Salt Solutions

**FIGURE 5:**
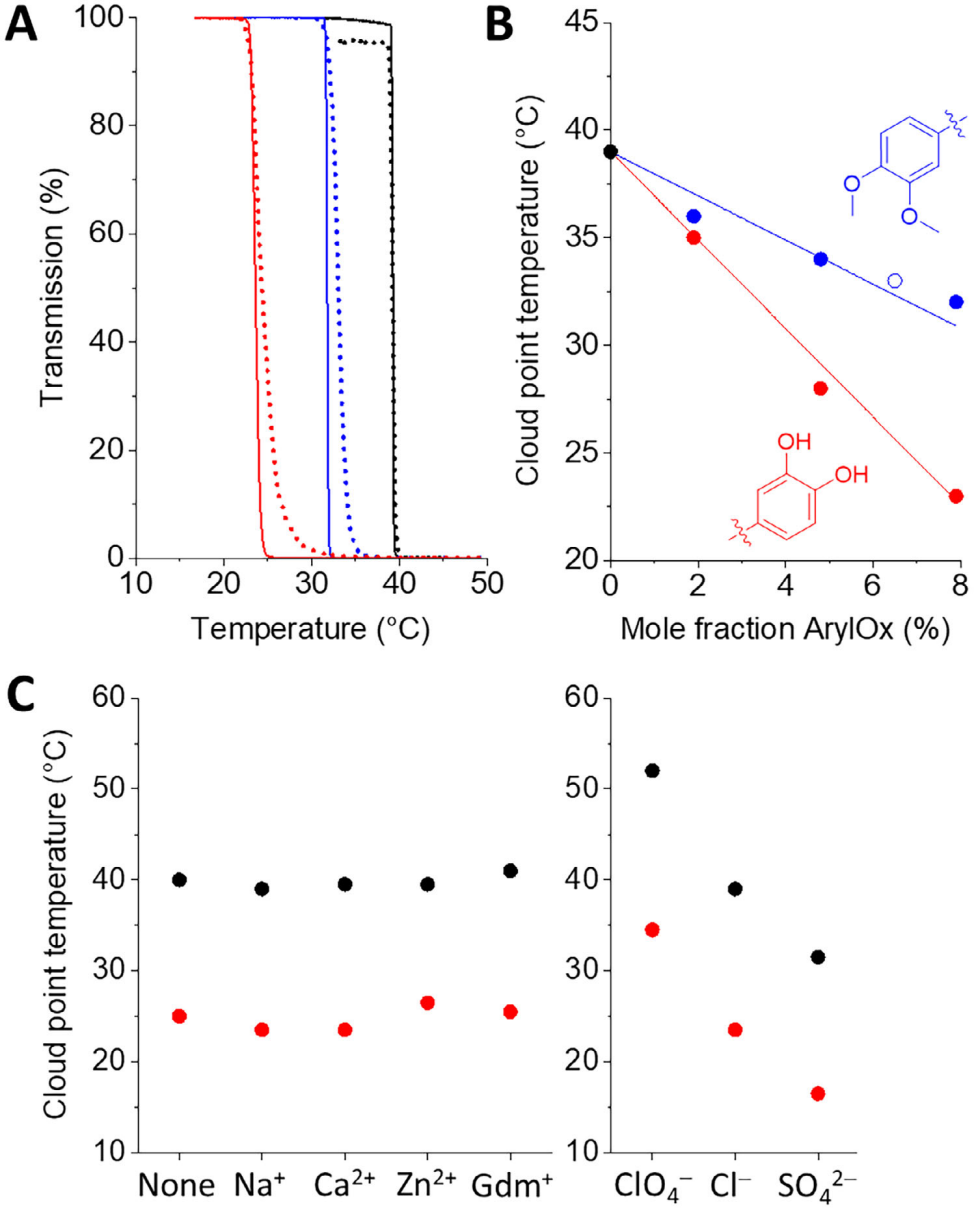
(A) Exemplary turbidity curves of 1 wt% PiPOx (black), **P_7.9_^OMe^** (blue), and **P_7.9_^OH^** (red) in physiological saline solution (heating: solid line, cooling: dotted line, 1 K min^−1^). (B) Cloud point temperatures of 1 wt% PiPOx (black circle), **P_1.9_^OMe^**, **P_4.8_^OMe^**, **P_6.5_^OMe^**, **P_7.9_^OMe^** (blue circles), **P_1.9_^OH^**, **P_4.8_^OH^**, and **P_7.9_^OH^** (red circles) saline solutions. (C) Cloud point temperatures of 1 wt% PiPOx (black circles) and **P_7.9_^OH^** (red circles) in 154 mM aqueous salt solutions (none = pure water without salt; Gdm^+^ = guanidinium); left: variation of cation (anion = Cl^−^) and right: variation of anion (cation = Na^+^).

Aqueous solutions (1 wt% polymer in physiological saline solution, 154 mM NaCl) of PiPOx, protected copolymers **P_6.5_^OMe^** (3.1.1), **P_1.9_^OMe^**, **P_4.8_^OMe^**, and **P_7.9_^OMe^** (3.1.2) and corresponding deprotected catechol‐containing copolymers **P_1.9_^OH^**, **P_4.8_^OH^**, and **P_7.9_^OH^** were analyzed by turbidimetric measurements (see Figure S15, Supporting Information) for the determination of cloud point temperature (*T*_CP_, at 80% transmission); results are summarized in Figure 5. The PiPOx solution exhibited a *T*_CP_ of 39°C and, notably, a sharp phase transition, and no hysteresis was observed between heating and cooling cycles (Figure 5A). The *T*_CP_s of the protected copolymer solutions were lower than those of PiPOx, as expected, attributable to the presence of additional hydrophobic 3,4‐dimethoxyphenyl side chains. The decrease of *T*_CP_ was found to correlate rather linearly with the mole fraction of DMPOx in the copolymer: *T*_CP_ = 36°C (**P_1.9_^OMe^**), 34°C (**P_4.8_^OMe^**), and 32°C (**P_7.9_^OMe^**) (Figure 5B). Notably, the saline solution of sample **P_6.5_^OMe^**, which contained some amounts of triethylammonium chloride (see above), showed a *T*_CP_ of 33°C. The corresponding deprotected copolymers, however, showed lower *T*_CP_s of 35°C (**P_1.9_^OH^**), 28°C (**P_4.8_^OH^**), and 24°C (**P_7.9_^OH^**). Again, the decrease of *T*_CP_ correlates linearly with the mole fraction of catechol units in the copolymer, however, the effect is even more pronounced than that appearing for the corresponding protected copolymers (Figure 5B). Hence, other than as expected, the deprotected catechol‐containing copolymers appear to be less hydrophilic than the protected precursor copolymers despite the presence of phenolic hydroxyl groups (see below).

Another interesting aspect is the effect of different salts on the cloud point temperature [39]. Here, we analyzed 1 wt% aqueous PiPOx and **P_7.9_^OH^** solutions containing different salts (154 mm), namely NaCl, CaCl_2_, ZnCl_2_, GdmCl, NaClO_4_, and Na_2_SO_4_; results are shown in Figure S16 (Supporting Information) and Figure 5C. Evidently, the *T*_CP_ remained virtually unchanged in the presence of the different cations (weak to strong chaotropes; Ca^2+^ and Zn^2+^ potentially forming metal−catecholate complexes [40]), i.e., PiPOx: *T*_CP_ 39–41°C and **P_7.9_^OH^**: *T*_CP_ 24–27°C, whereas it was significantly shifting with different anions. Compared to the *T*_CP_ in saline solution, the *T*_CP_ was higher in NaClO_4_ solution (PiPOx: Δ*T*_CP_ +13°C, **P_7.9_^OH^**: Δ*T*_CP_ +11°C) and it was lower in Na_2_SO_4_ solution (PiPOx: Δ*T*_CP_ −8°C, **P_7.9_^OH^**: Δ*T*_CP_ −7°C). Interestingly, Δ*T*_CP_ is very similar for both PiPOx and **P_7.9_^OH^** solutions. The observed salting in of the (co)poly(2‐isopropyl‐2‐oxazoline)s with ClO_4_^−^ (chaotrope) and the salting out with SO_4_^2−^ (kosmotrope) is in line with the Hofmeister series (see also [39]). Still, however, the underlying mechanism is not clear.

Notably, we also examined PiPOx and **P_7.9_^OH^** in 154 mm FeCl_3_ solution. The PiPOx/FeCl_3_ solution exhibited a *T*_cp_ of 40°C (Figure S16, Supporting Information), indicating that the solubility of PiPOx was also not affected by Fe^3+^ ions (see above). The *T*_cp_ of the **P_7.9_^OH^**/FeCl_3_ solution, on the other hand, could not be measured due to the immediate appearance of an intense green‐blue color (*λ*_max_ ~ 680 nm, see Figure S17, Supporting Information), attributable to the presence of mono‐catechol−Fe^3+^ complexes [41, 42] (which however is clearly different to 1,2‐dihydroxybenzene/FeCl_3_ with *λ*_max_ ~ 550 nm, Figure S17, Supporting Information). It is also worth noting that the complexation of Fe^3+^ ions occurred spontaneously in acidic aqueous medium (pH ∼4.5) without the need to actively deprotonate the catechol units with a base [43].

However, independent of the observed salt effects, the *T*_CP_ of the catechol‐containing **P_7.9_^OH^** solutions is consistently lower than that of PiPOx and protected copolymer solutions, suggesting a decreased hydrophilicity of **P_7.9_^OH^**. Raman spectroscopy was used to understand on the molecular level what happens due to the hydration of polymers. The hydration level of polymers can either be characterized by controlling the concentration of polymer in solution or by checking the changes in spectra during dehydration [44, 45, 46]. We selected the second option since the amount of sample required is small, in contrast to the first option. First, we analyzed the freeze‐dried samples of PiPOx, **P_7.9_^OMe^**, and **P_7.9_^OH^** (see the Raman spectra in Figure S18, Supporting Information). The observed peaks at 1090 and 1190 cm^−1^ are related to skeletal ν(C‐C), the peak at 1260 cm^−1^ to ν(C‐N), the doublet at 1454 and 1481 cm^−1^ to CH_3_ deformations, and the intense peak at 1630 cm^−1^ to the amide I band. The catechol units were observed at 1610 cm^−1^ related to the C═C vibrations of the aromatic ring; the methoxy groups of protected catechol arise at 2840 cm^−1^ [47]. With increasing irradiation time under ambient atmosphere, we recognized photooxidation of the catechol to quinone by the appearance of a peak at 1725 cm^−1^ related to C═O stretching (see Figure S19, Supporting Information). This process not only occurred for **P_7.9_^OH^** but also to a lesser extent for the protected **P_7.9_^OMe^**, showing that the applied Raman laser irradiation power was seemingly enough to break O─CH_3_ bonds.

**FIGURE 6:**
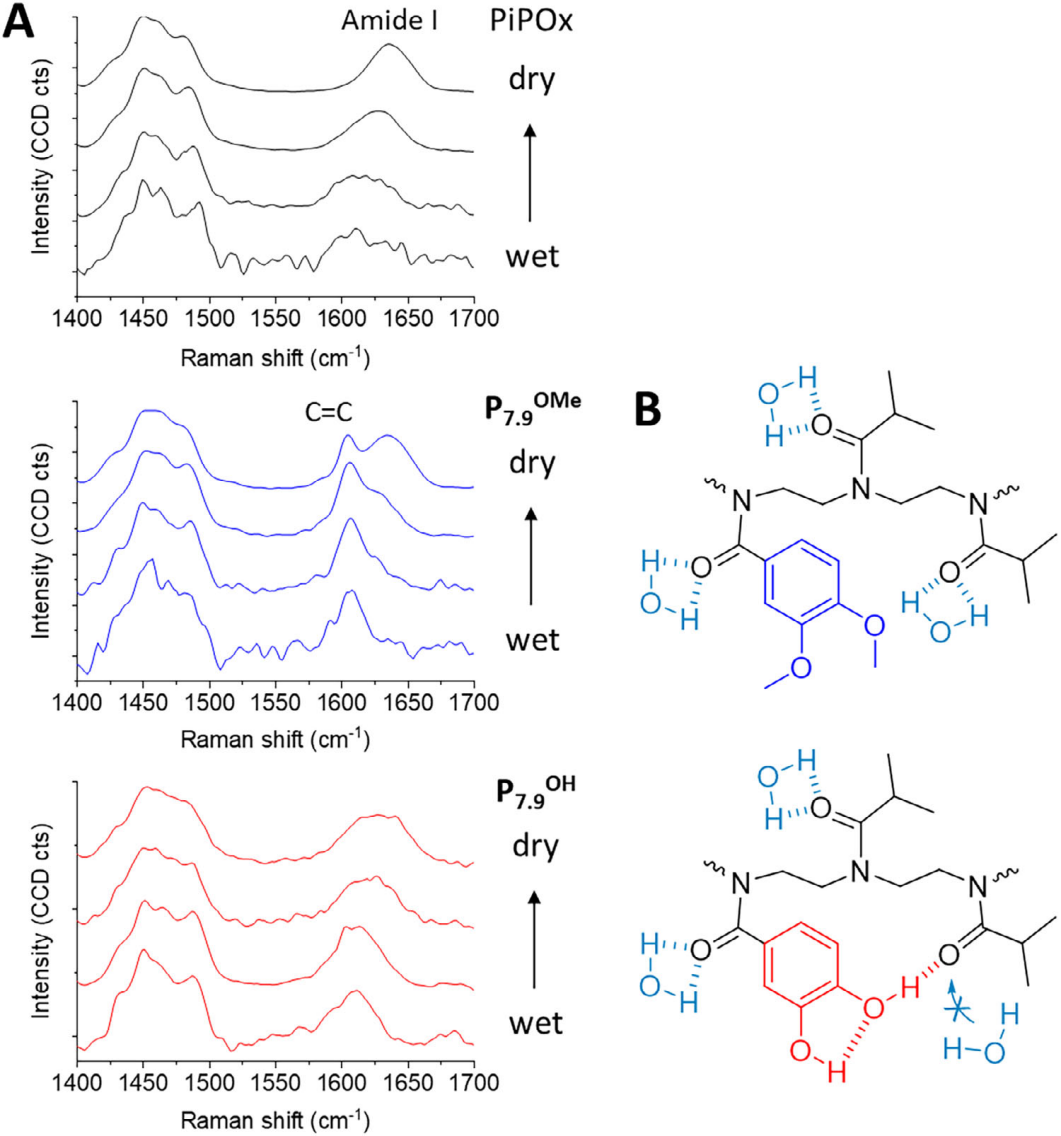
(A) Raman spectra (1400‐1700 cm^−1^ region) of PiPOx, **P_7.9_^OMe^**, and **P_7.9_^OH^** (top to bottom) obtained during passive dehydration of the samples. (B) Tentative illustration of hydrogen bonding interactions of polymer amide side chains with catechol and water.

Upon addition of water, the first observation was that most of the spectrum of PiPOx did not change, i.e., the peaks remained in the same position with overall similar intensity (see Figure 6A and Figure S20, Supporting Information). The most significant change was related to the peak associated with the amide group at 1635 cm^−1^. In all cases, we observed an intensity decrease and the band appeared to split into two weaker bands at ∼1615 and ∼1690 cm^−1^. The appearance of these two bands upon hydration is related to the interaction of the polymer with water molecules, where a certain amount of amide is less rigid (∼1615 cm^−1^) and a lower amount is more rigid due to intramolecular interactions (∼1690 cm^−1^). In other words, water is interacting strongly with the PiPOx amide groups in the polymer via hydrogen bonding [44, 48]. Using the second derivative of the signal (Figure S18, Supporting Information), we could better assign the peak positions and found even three peaks: one at ∼1610 cm^−1^ related to the solvated polymer, another at 1630 cm^−1^ related to hydrogen bonding either with water or intramolecular hydrogen bonding, and a peak at 1690 cm^−1^ related to strong intramolecular interactions between side chains [48].

For **P_7.9_^OMe^** and **P_7.9_^OH^**, the band at ∼1615 cm^−1^ is not clearly observed, also not in the second derivative, due to the strong C═C vibrations of the aromatic ring and the overlap with the solvated amide band (see Figure 6A; Figure S20, Supporting Information). Intriguingly, the peak at 1610 cm^−1^ is broader for the **P_7.9_^OH^** (FWHM ∼35 cm^−1^) compared to the **P_7.9_^OMe^** (FWHM ∼22 cm^−1^). We attribute this difference to the interaction of catechol hydroxyl groups with the polymer amide side chains via hydrogen bonding (compare to hydrogen bonding in catecholylamides [49] and catechol functionalized ε‐polylysine/polyacrylamide hydrogels [50]). Because of the interactions between the two functional groups, the vibrations couple and appear as a single component in the spectra of **P_7.9_^OH^**, whereas for **P_7.9_^OMe^** the amide band is still observed at 1640 cm^−1^ (see Figure S20, Supporting Information). We therefore propose that for PiPOx and **P_7.9_^OMe^** water can solvate all amide side chains, keeping its high solubility, while for **P_7.9_^OH^** the catechol is interacting with the amide backbone via hydrogen bonding, hindering the hydration of the polymer (as illustrated in Figure 6B). In other words, **P_7.9_^OH^** can bind lesser amounts of water molecules than PiPOx or **P_7.9_^OMe^**, thus explaining its decreased hydrophilicity observed in the turbidity measurements (see above).

## 4 Conclusions

A small series of catechol‐containing poly(2‐isopropyl‐2‐oxazoline)s (1.9–7.8 mol% catechol) was synthesized by either functionalization of partially hydrolyzed PiPOx with 3,4‐dimethoxybenzoyl chloride or by microwave‐assisted statistical copolymerization of iPOx and 2‐(3,4‐dimethoxyphenyl)‐2‐oxazoline (DMPOx) at 140°C. The ideal copolymerization yielded slightly gradient copolymers (reactivity parameters: *r*_1_ (iPOx) = 2.6 and *r*_2_ (DMPOx) = 0.46). Quantitative demethylation of protected catechol units was achieved by treatment with BBr_3_ (using a large excess of the Lewis acid of 4 equiv. with respect to amide groups) at room temperature without noticeable formation of quinones.

PiPOx and all copolymer samples were soluble at 1 wt% in aqueous 154 mm salt solutions at room temperature and exhibited thermoresponsive LCST behavior. The cloud point temperatures decreased in the order PiPOx (*T*_CP_ 39°C) > protected catechol‐containing PiPOx > catechol‐containing PiPOx, also depending on the amount of catechol units in the copolymer. While the *T*_CP_ remained virtually unchanged in the presence of different cations (Na^+^, Ca^2+^, Zn^2+^, and Gdm^+^), it was strongly affected by anions (ClO_4_^−^, chaotrope, and SO_4_^2−^, kosmotrope), which is in line with the Hofmeister series. The lower hydrophilicity of the catechol‐containing PiPOx as compared to the protected precursor copolymer (and PiPOx) is suggested to be due to hydrogen bonding interactions between catechol and tertiary amide units of the polymer backbone, as indicated by Raman spectroscopy, which decreases the overall hydration level of the polymer.

The ability of the catechol‐containing PiPOx to bind to metal ions in aqueous solution was exemplarily demonstrated for ferric ions. UV–vis absorption spectroscopy indicated the spontaneous formation of mono‐catechol−Fe^3+^ complexes (*λ*_max_ ~ 680 nm) along the polymer chain.

In the future, we plan to further examine the thermoresponsive solution behavior of the catechol‐containing PiPOx copolymers, with a particular focus on crystallization above the cloud point temperature, and extend the complexation studies to a broader variety of metal ions, including structural analysis of catechol‐metal ion complexes and determination of association constants, aiming, for instance, for application in water purification. We also envision using the catechol‐containing PiPOx as an additive for biomineralization and for the coating of metal nanoparticles and surfaces.

## Conflicts of Interest

The authors declare no conflict of interest.

## Supporting information




**Supporting File**: marc70113‐sup‐0001‐SuppMat.pdf.

## Data Availability

The data that support the findings of this study are available in the supplementary material of this article.
