# Supplementary material for: Catechol‐Containing Poly(2‐isopropyl‐2‐oxazoline): Synthesis and Thermoresponsive Behavior in Aqueous Salt Solutions
Source: Macromol Rapid Commun. 2025 Oct 30;47(15):e00719. doi: 10.1002/marc.202500719 (PMC13435113; doi:10.1002/marc.202500719)
Supplement: Supplementary file 1 [file MARC-47-e00719-s001.pdf]

Supporting Information

to

Catechol-containing copoly(2-isopropyl-2-oxazoline): Synthesis and thermoresponsive behavior in aqueous salt solutions

Niclas Madaj, Nils Lüdecke, Sergio Kogikoski Jr., Ilko Bald, and Helmut Schlaad*

Institute of Chemistry, University of Potsdam, Karl-Liebknecht Str. 24-25,
14476 Potsdam, Germany

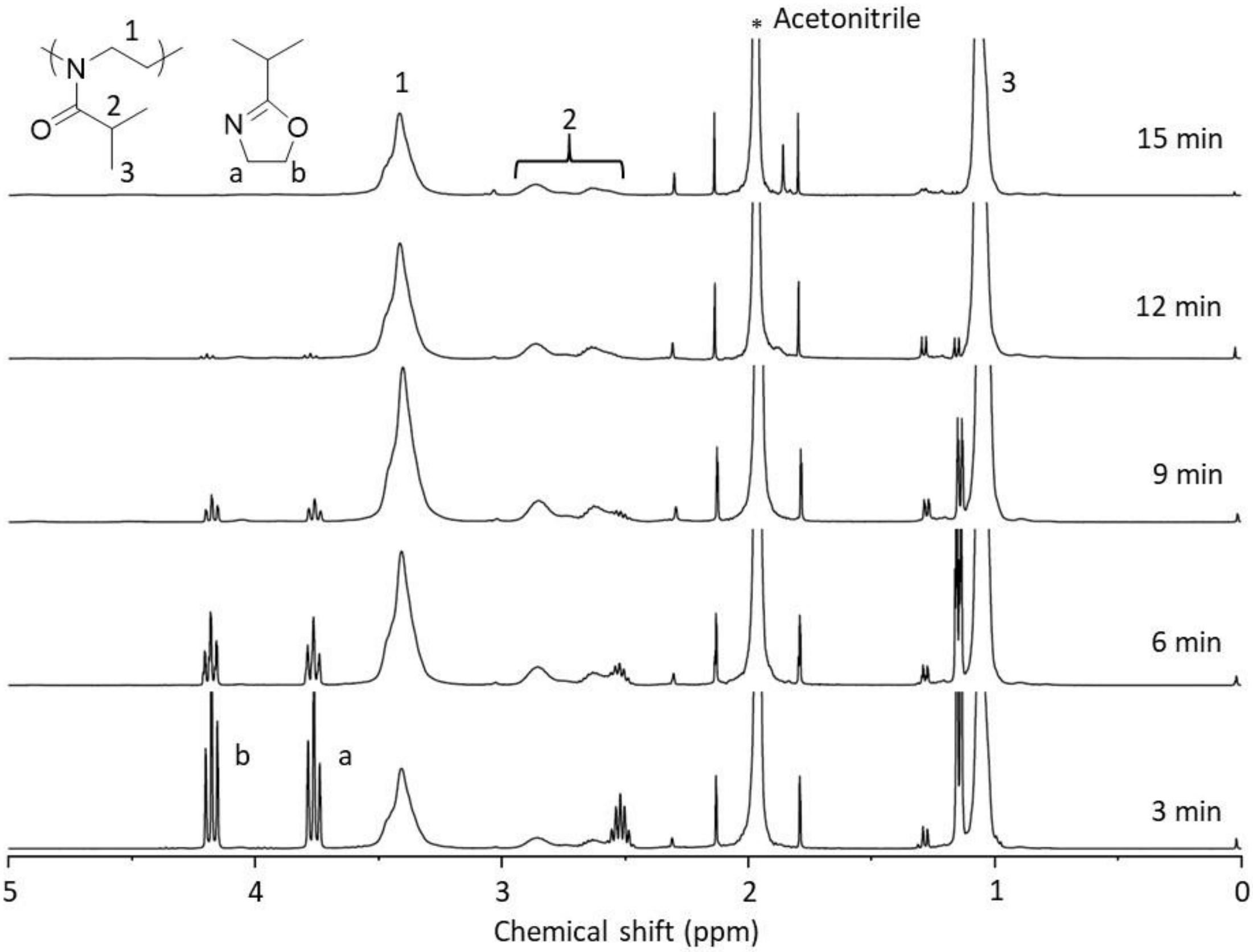

Figure S1. ^{1}H NMR (400 MHz, $CDCl_3$) spectra of crude reaction mixtures obtained during the microwave-assisted polymerization of 2-isopropyl-2-oxazoline (iPOx) in acetonitrile solution at 140 °C.

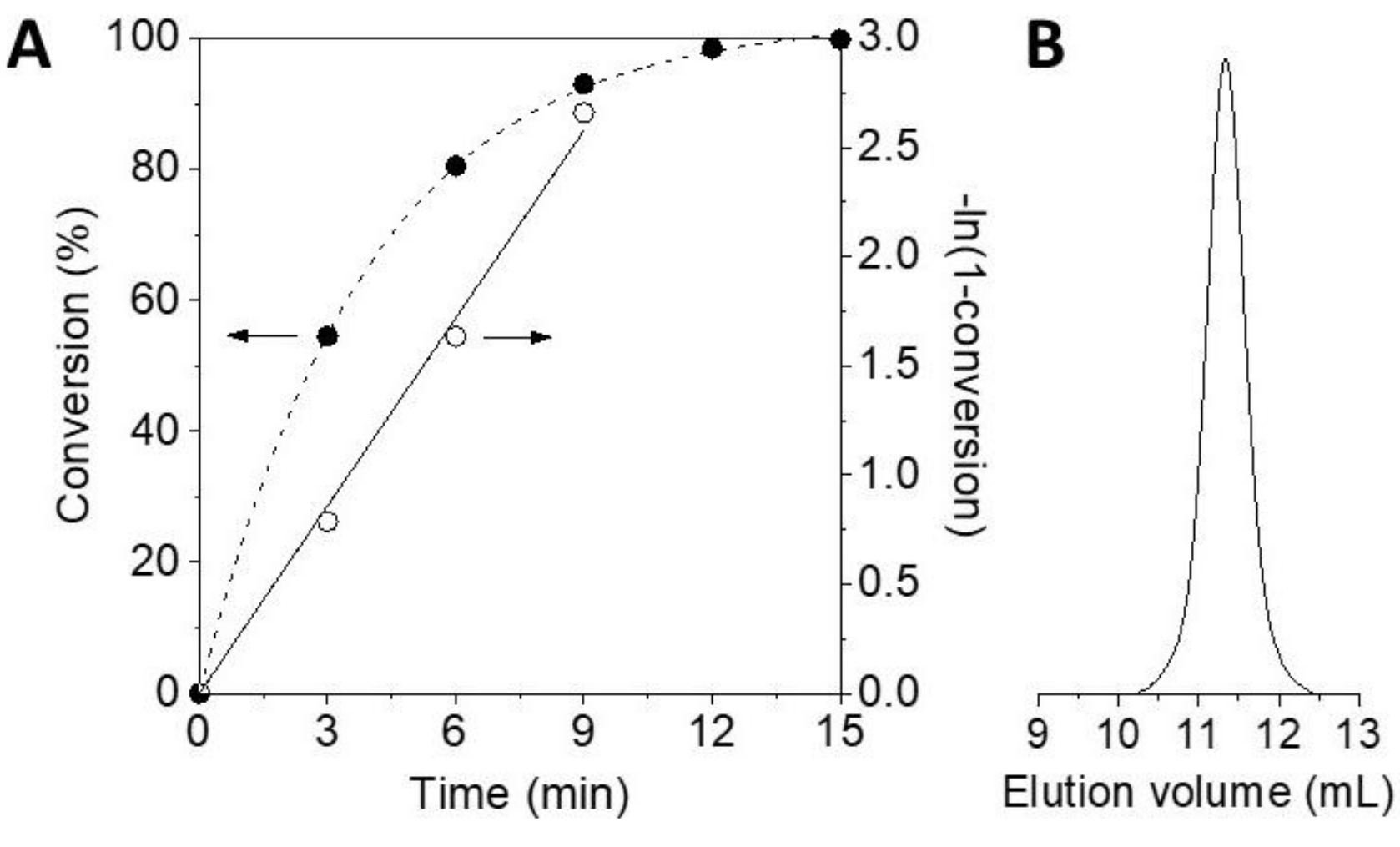

Figure S2. (A) Time-conversion plot for the microwave-assisted CROP of 2-isopropyl-2-oxazoline ($[iPOx]_0$ = 1.82 M and $[iPOx]_0/[MeOTos]_0$ = 100) in acetonitrile solution at 140 °C. (B) Exemplary SEC-RI trace (eluent: NMP) of a poly(2-isopropyl-2-oxazoline) sample obtained at full monomer conversion.

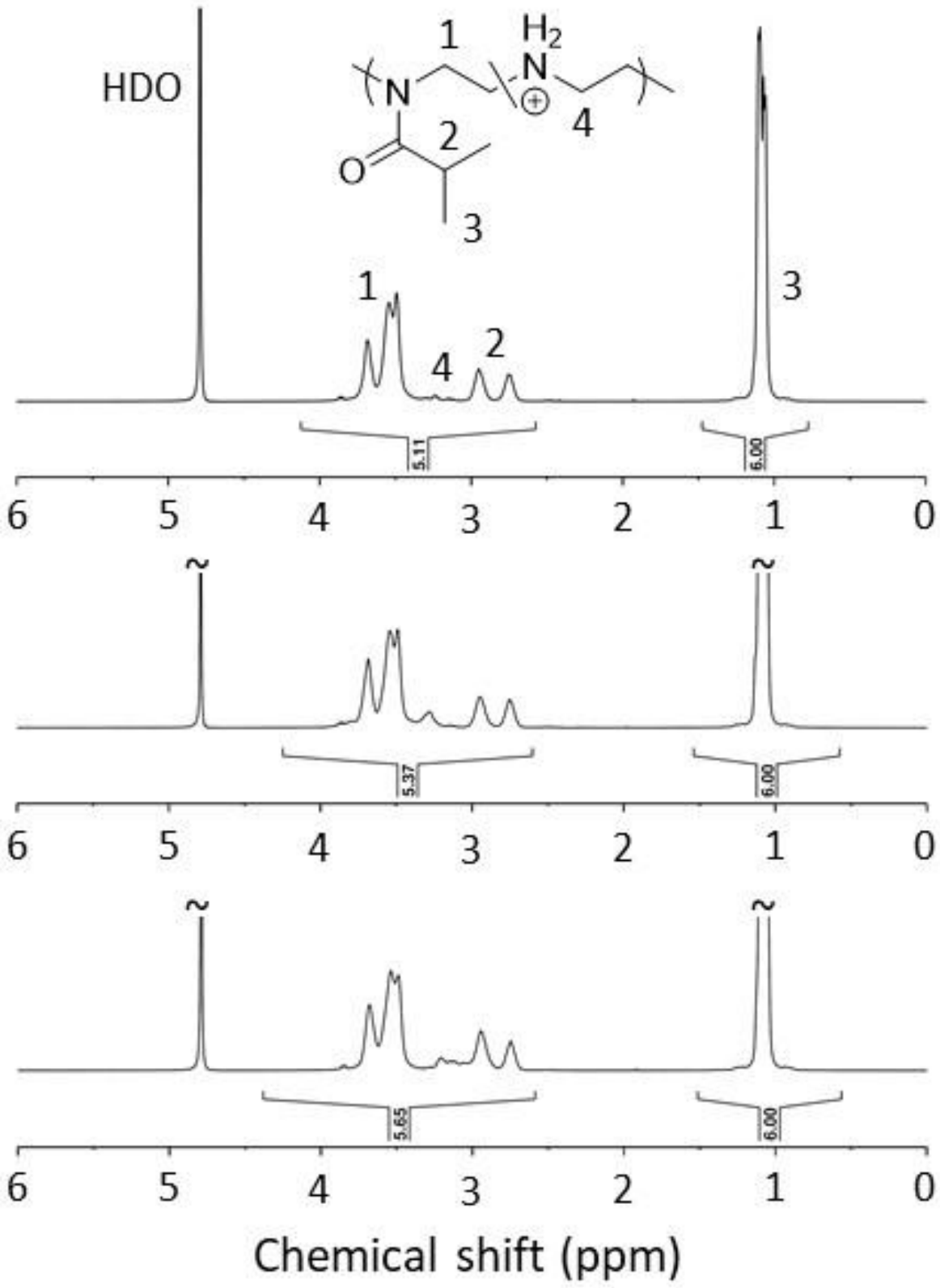

Figure S3. Exemplary ^{1}H NMR (400 MHz, D_2O) spectra of partially hydrolyzed PiPOx samples obtained by microwave-assisted hydrolysis of PiPOx in 6M HCl solution at 100 °C after a reaction time of 5, 15, and 25 min (top to bottom).

Determination of the degree of hydrolysis (x) of PiPOx by ^{1}H NMR spectroscopy (Figure S3): Peak integral A at δ 2.6-3.9 ppm contains 5H of non-hydrolyzed PiPOx units and 4H of EI units, thus $A = 5(1-x) + 4x$, and peak integral B at 1.1 ppm contains 6H of methyl groups from the isopropyl side chain of PiPOx, thus $B = 6(1-x)$.

$$\Rightarrow \frac{5(1-x)+4x}{6(1-x)} = \frac{A}{B}$$

$$\Rightarrow \frac{5}{6} + \frac{4x}{6(1-x)} = \frac{A}{B} \qquad | \cdot 6 \quad | -5$$

$$\Rightarrow \frac{4x}{1-x} = 6\frac{A}{B} - 5 \qquad | \, ()^{-1}$$

$$\Rightarrow \frac{1}{4x} - \frac{1}{4} = \frac{1}{6\frac{A}{B}-5} \qquad | \cdot 4 \quad | +1$$

$$\Rightarrow \frac{1}{x} = 1 + \frac{4}{6\frac{A}{B}-5} \qquad | \, ()^{-1}$$

$$\Rightarrow x = \left(1 + \frac{4}{6\frac{A}{B}-5}\right)^{-1}$$

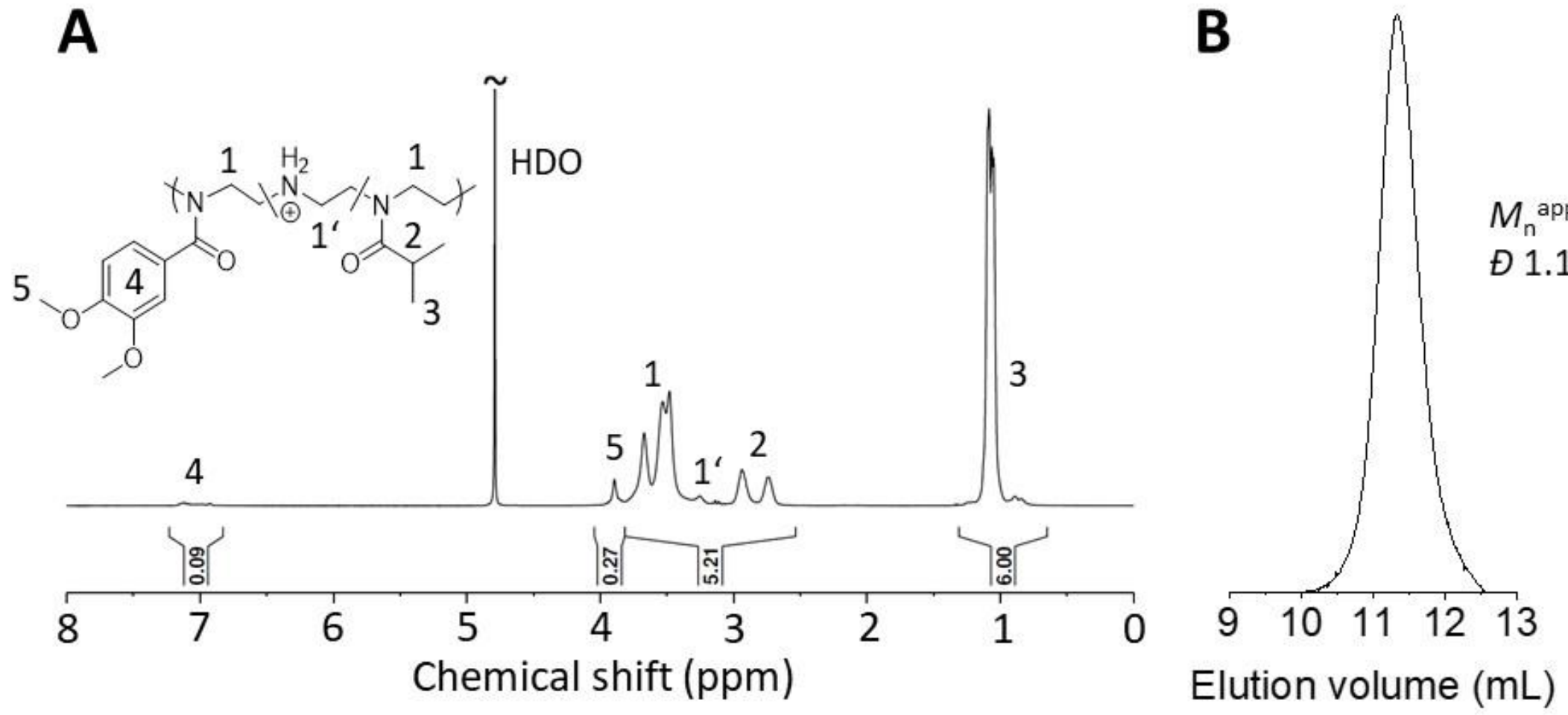

Figure S4. (A) ^{1}H NMR (400 MHz, D_2O) spectrum and (B) SEC-RI trace (eluent: NMP) of the copolymer obtained by functionalization of partially hydrolyzed PiPOx (degree of hydrolysis 6.5%) with DMBzCl using pyridine as a base.

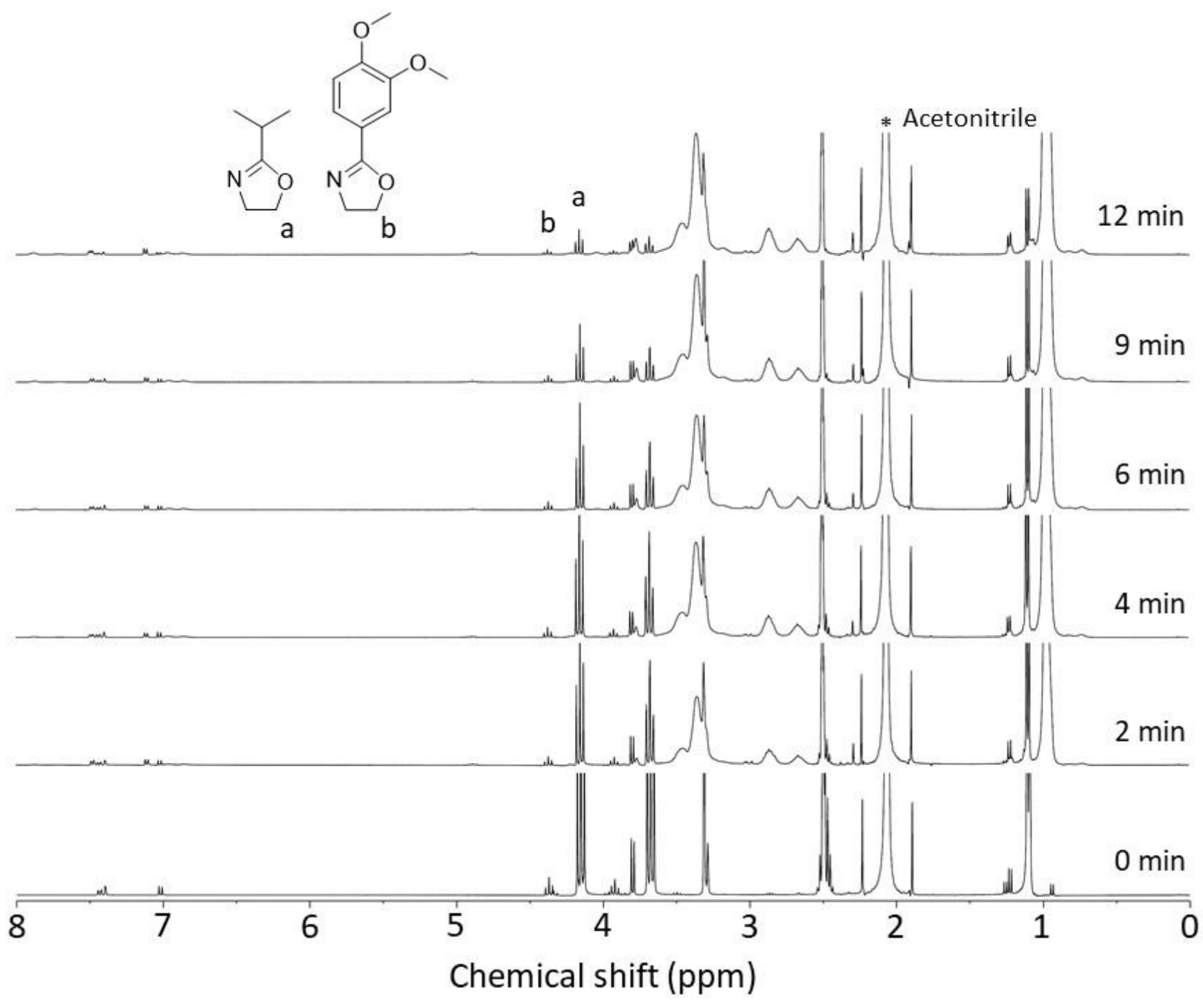

Figure S5. ^{1}H NMR (400 MHz, DMSO-d_6) spectra of crude reaction mixtures obtained during the microwave-assisted copolymerization of 2-isopropyl-2-oxazoline and 2-(3,4-dimethoxy-phenyl)-2-oxazoline (98:2) in acetonitrile solution at 140 °C.

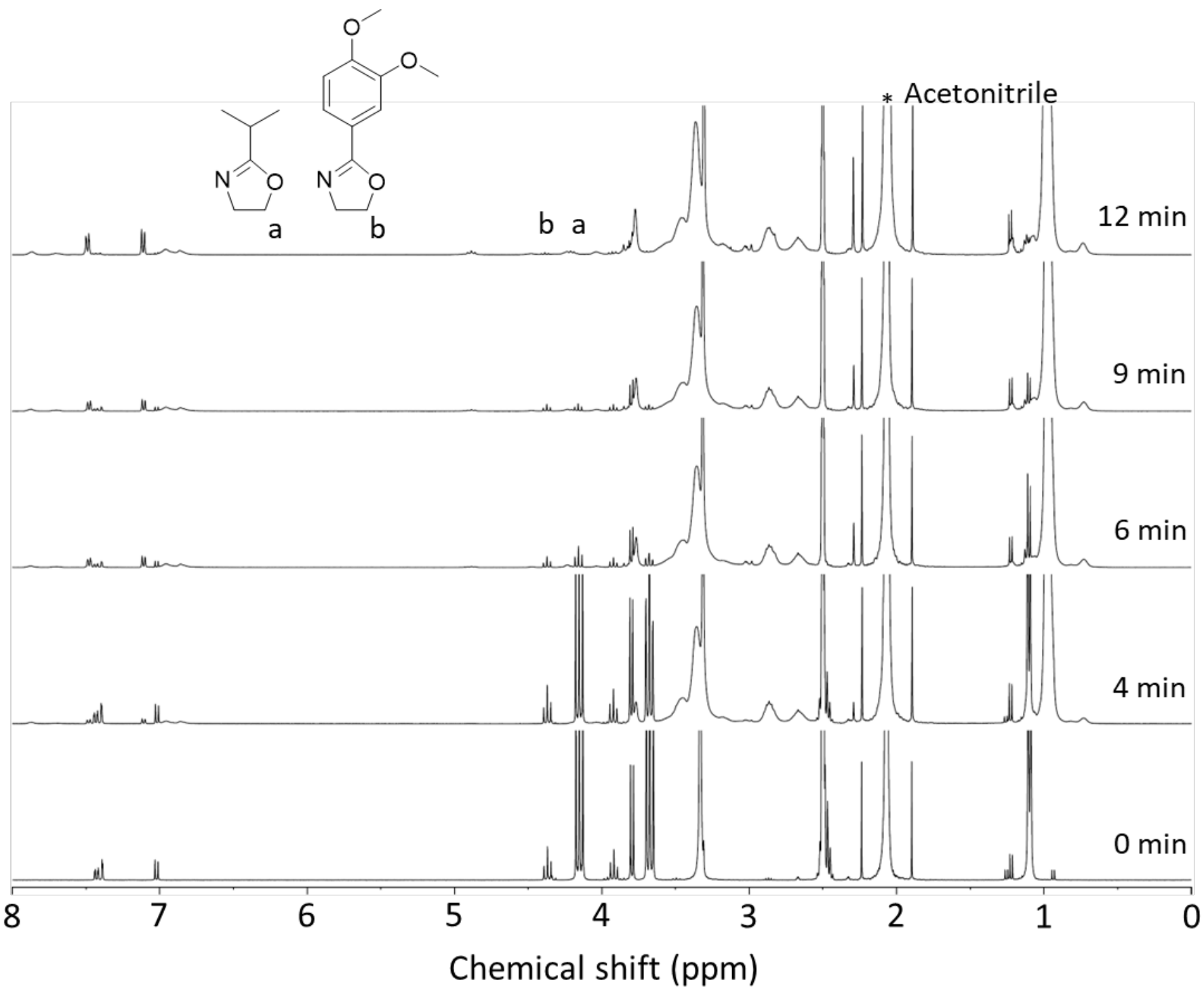

Figure S6. ^{1}H NMR (400 MHz, DMSO-d$_6$) spectra of crude reaction mixtures obtained during the microwave-assisted copolymerization of 2-isopropyl-2-oxazoline and 2-(3,4-dimethoxy-phenyl)-2-oxazoline (95:5) in acetonitrile solution at 140 °C.

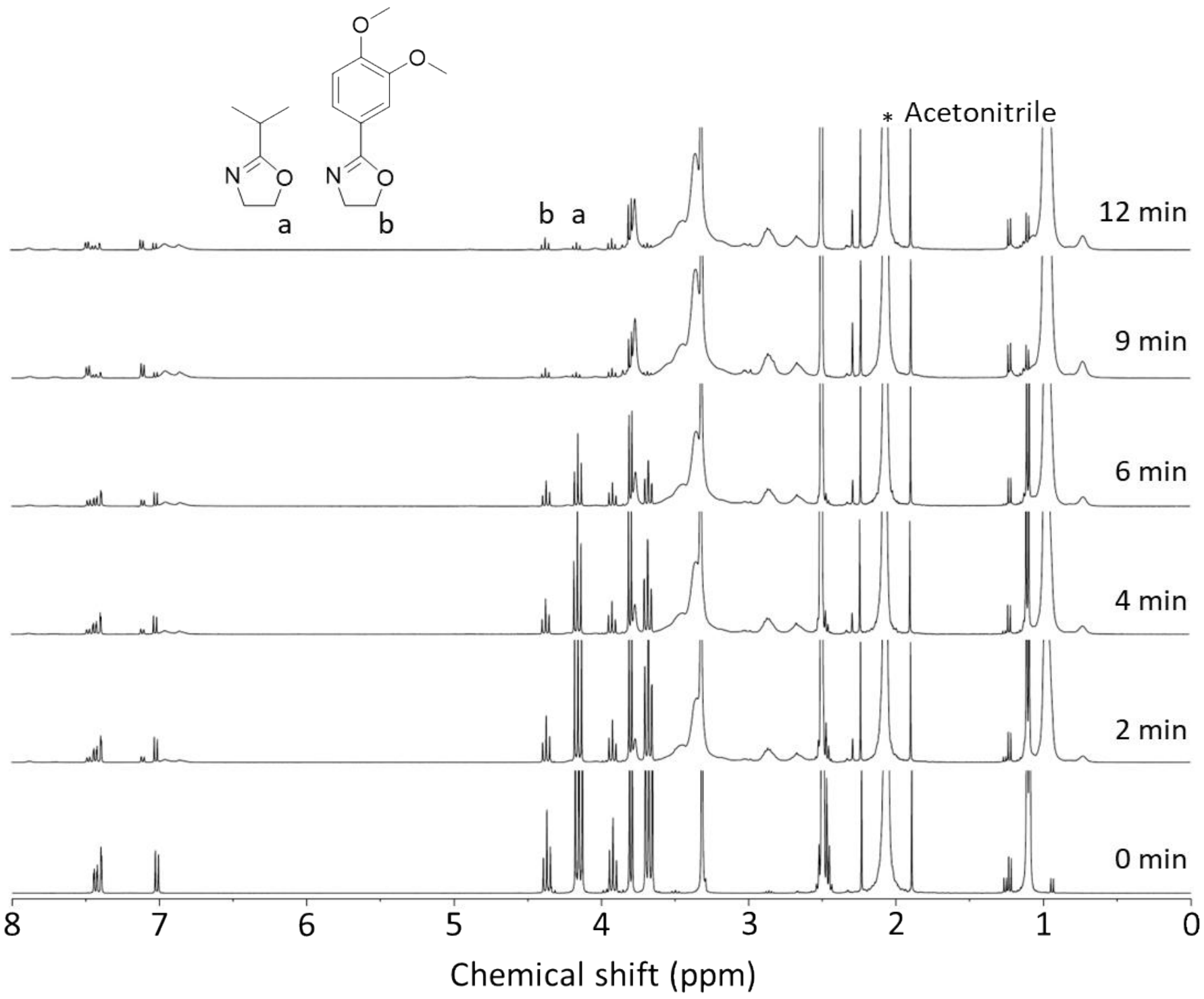

Figure S7. ^{1}H NMR (400 MHz, DMSO-d$_6$) spectra of crude reaction mixtures obtained during the microwave-assisted copolymerization of 2-isopropyl-2-oxazoline and 2-(3,4-dimethoxy-phenyl)-2-oxazoline (90:10) in acetonitrile solution at 140 °C.

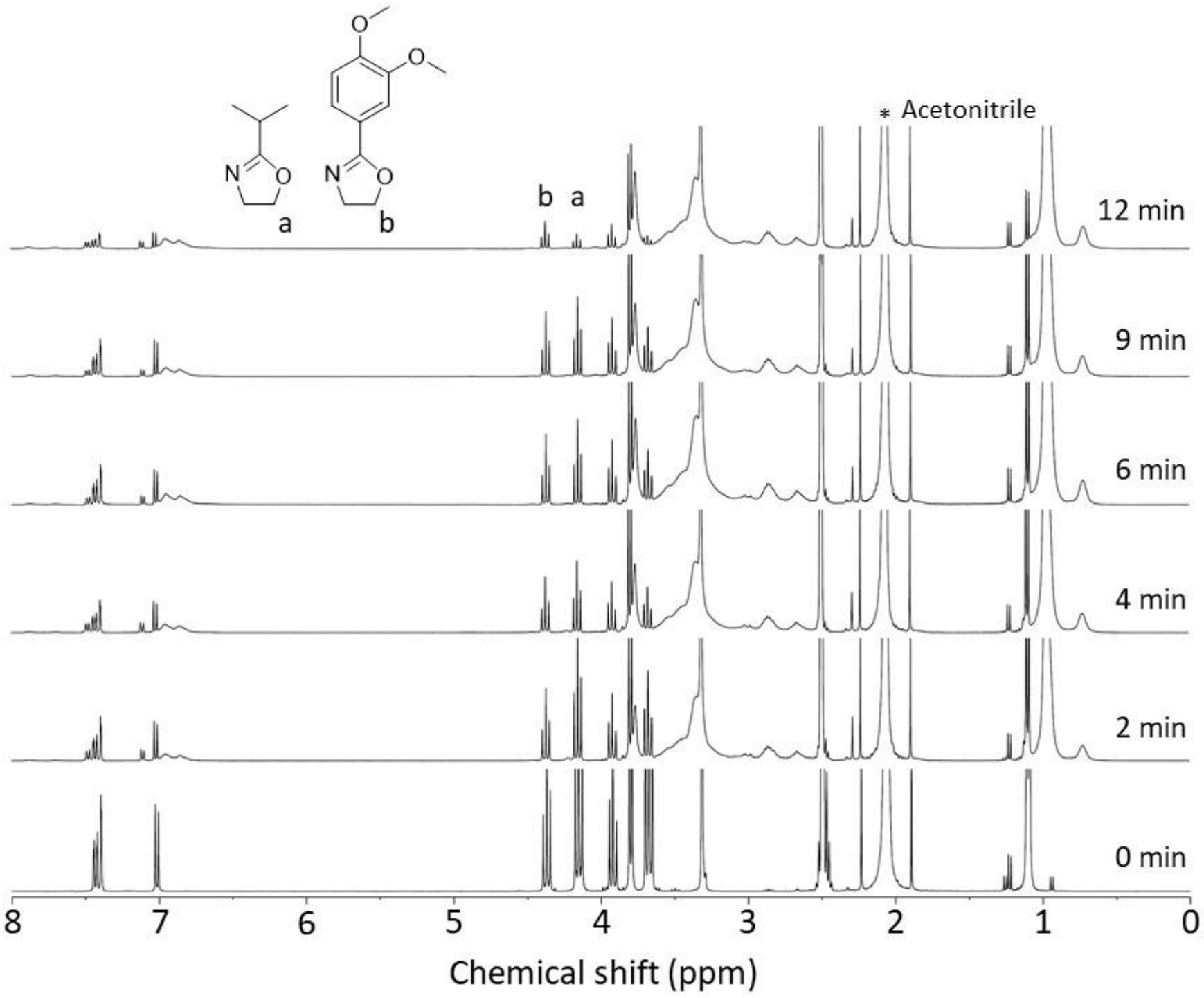

Figure S8. ^{1}H NMR (400 MHz, DMSO-d$_6$) spectra of crude reaction mixtures obtained during the microwave-assisted copolymerization of 2-isopropyl-2-oxazoline and 2-(3,4-dimethoxy-phenyl)-2-oxazoline (80:20) in acetonitrile solution at 140 °C.

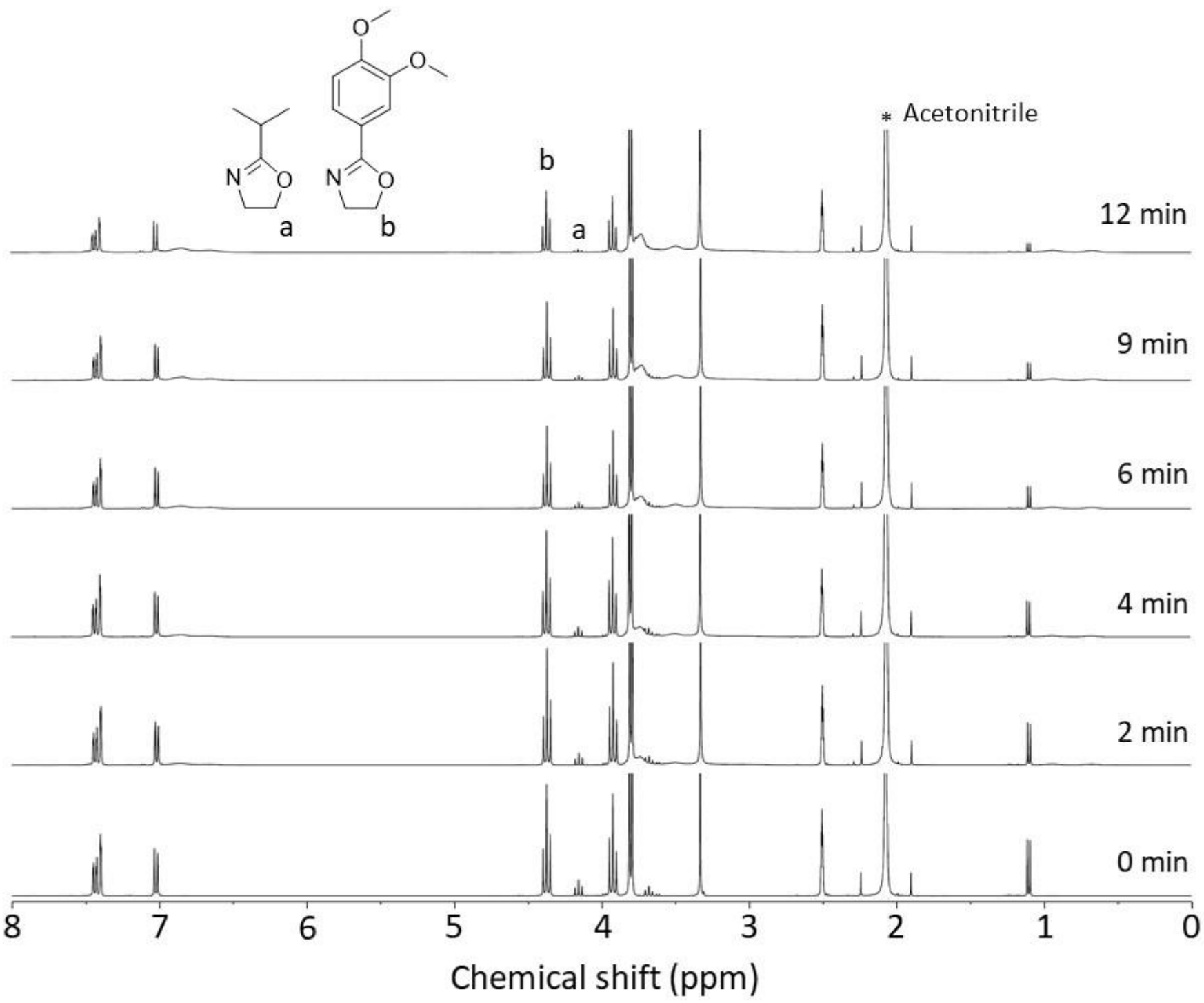

Figure S9. ^{1}H NMR (400 MHz, DMSO-d$_6$) spectra of crude reaction mixtures obtained during the microwave-assisted copolymerization of 2-isopropyl-2-oxazoline and 2-(3,4-dimethoxy-phenyl)-2-oxazoline (10:90) in acetonitrile solution at 140 °C.

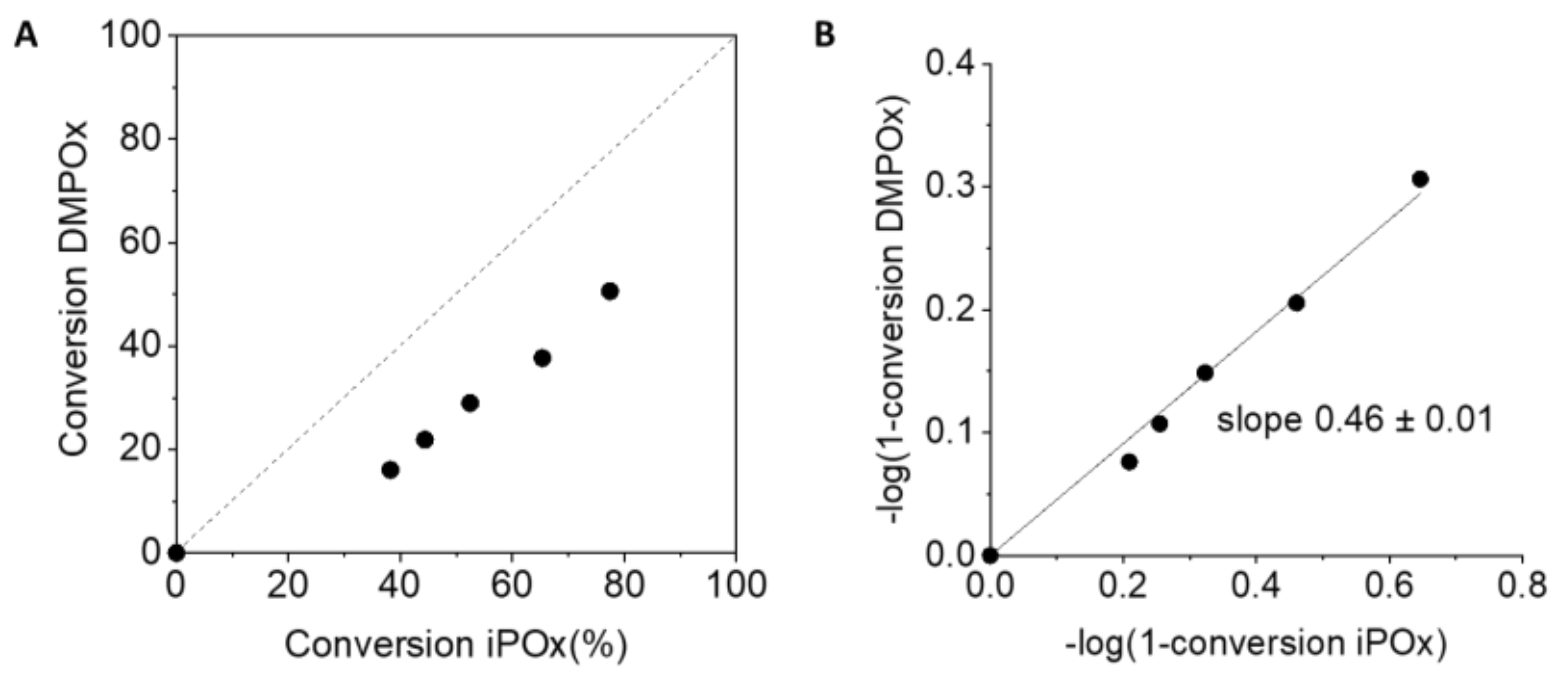

Figure S10. (A) Monomer conversions for the microwave-assisted copolymerization of a mixture of 2-(3,4-dimethoxyphenyl)-2-oxazoline (DMPOx) and 2-isopropyl-2-oxazoline (iPOx) 90:10 in acetonitrile solution at 140 °C and (B) corresponding Jaacks plot.

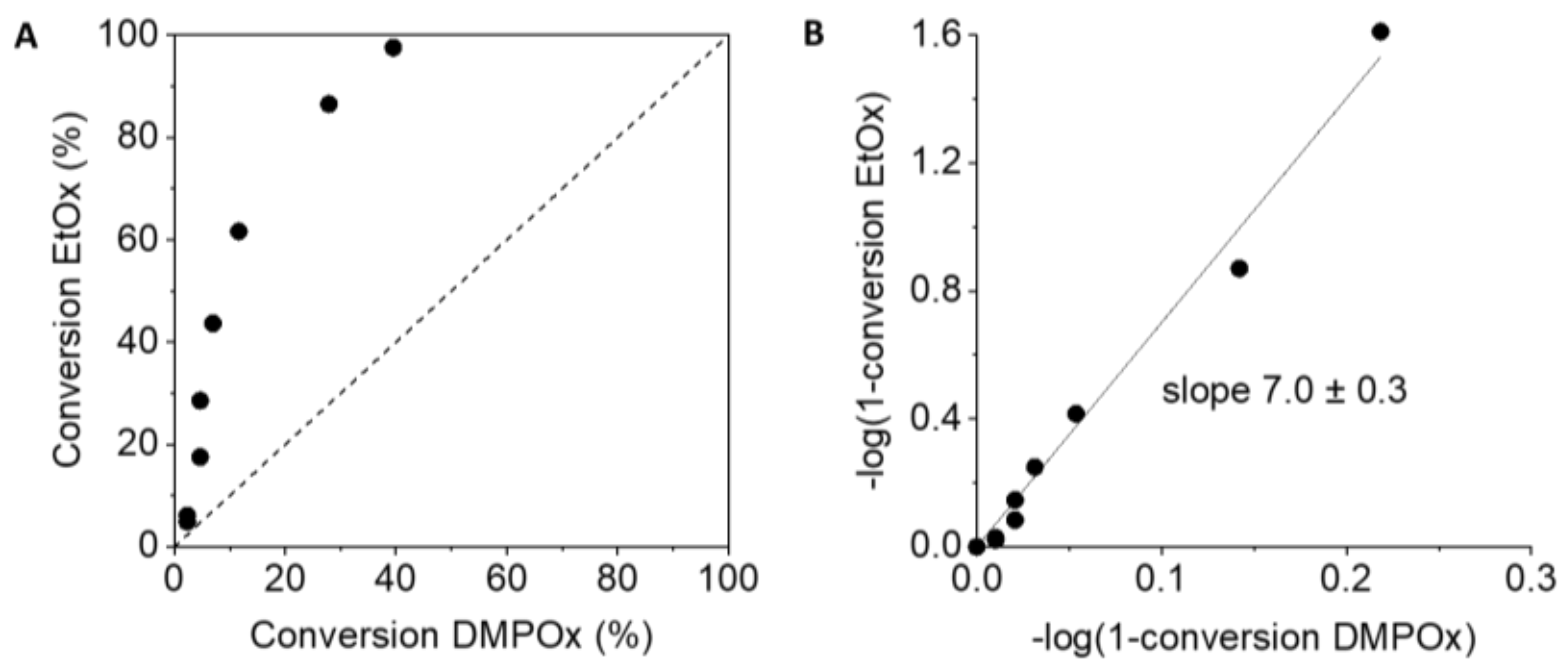

Figure S11. (A) Monomer conversions for the microwave-assisted copolymerization of mixture of 2-ethyl-2-oxazoline (EtOx) and 2-(3,4-dimethoxyphenyl)-2-oxazoline (DMPOx) 90:10 in acetonitrile solution at 100 °C (data taken from N. Lüdecke, H. Schlaad, *Polym. Chem.* **2021**, *12*, 5310) and (B) corresponding Jaacks plot.

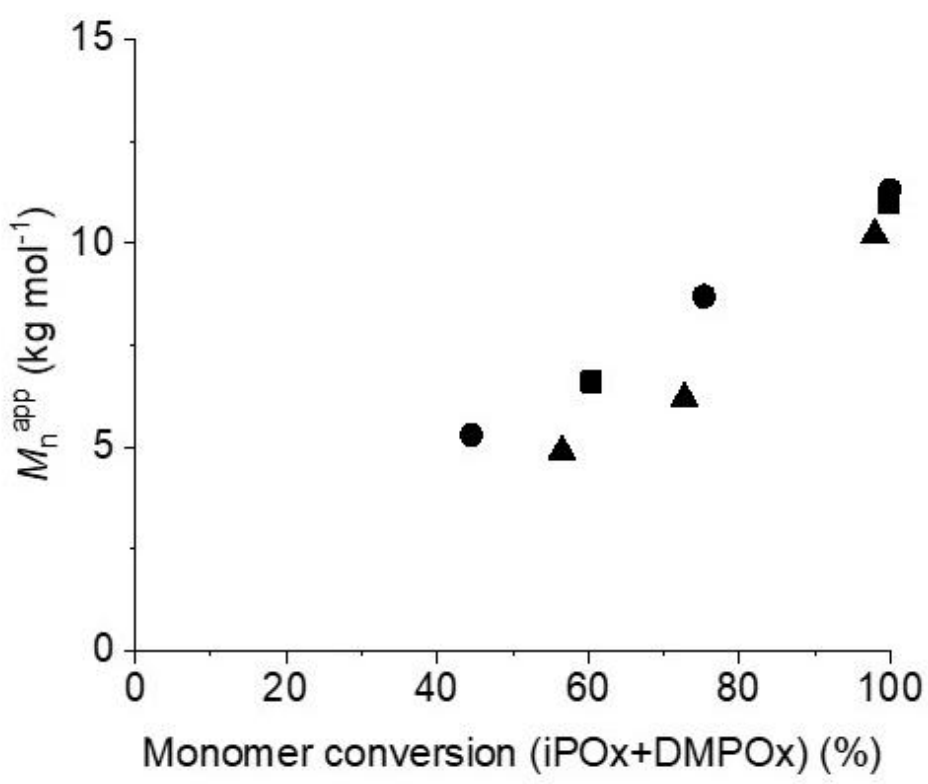

Figure S12. Evolution of copolymer molar mass (M_n^{app}, by SEC) with monomer conversion during the microwave-assisted copolymerization of 2-isopropyl-2-oxazoline (iPOx) and 2-(3,4-dimethoxy-phenyl)-2-oxazoline (DMPOx) 98:2 (circles), 95:5 (squares), and 90:10 (triangles).

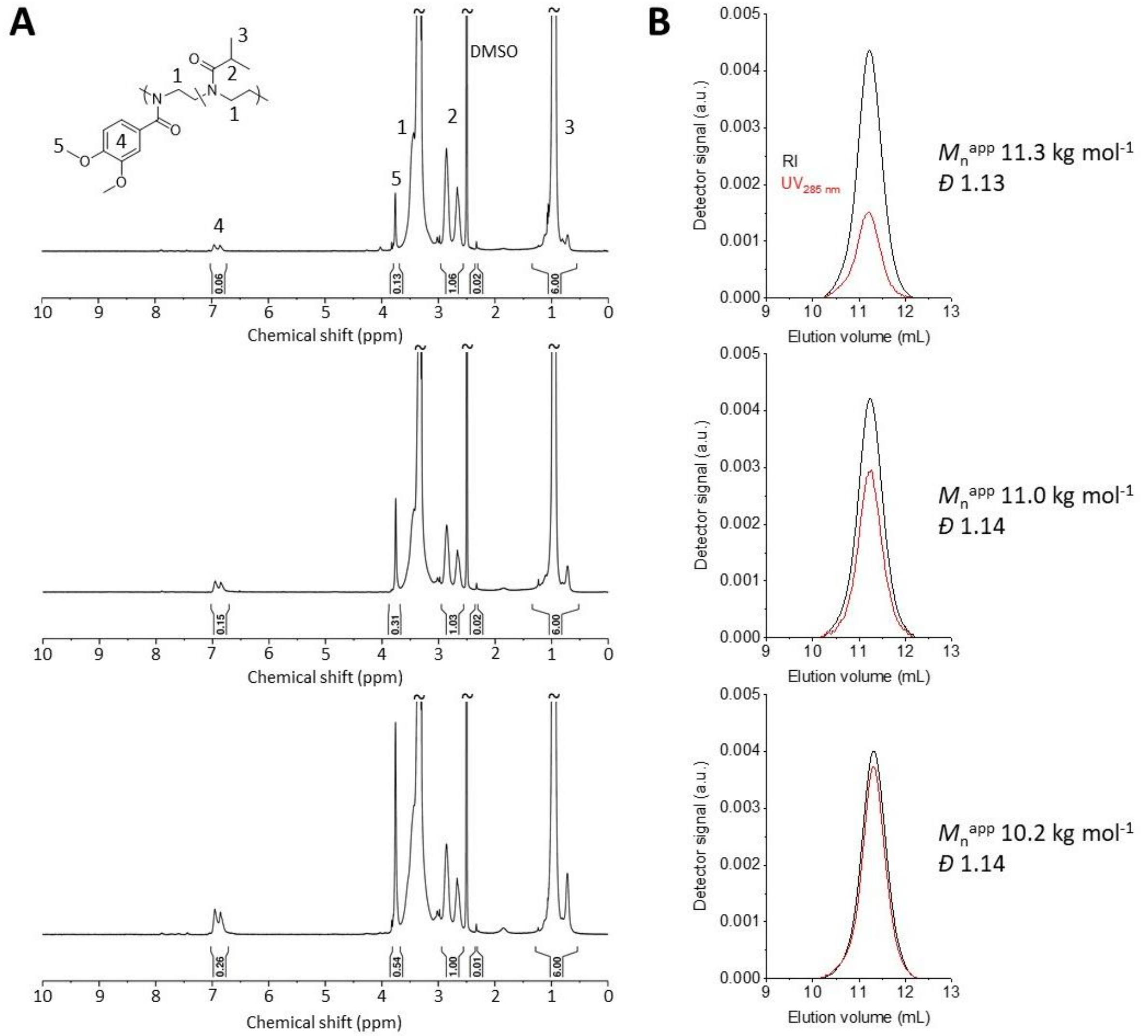

Figure S13. (A) ^{1}H NMR (400 MHz, DMSO-d$_6$) spectra and (B) SEC-RI/UV$_{285\ nm}$ traces (eluent: NMP) of the copolymers $\mathbf{P_{1.9}^{OMe}}$, $\mathbf{P_{4.8}^{OMe}}$, and $\mathbf{P_{7.9}^{OMe}}$ (top to bottom).

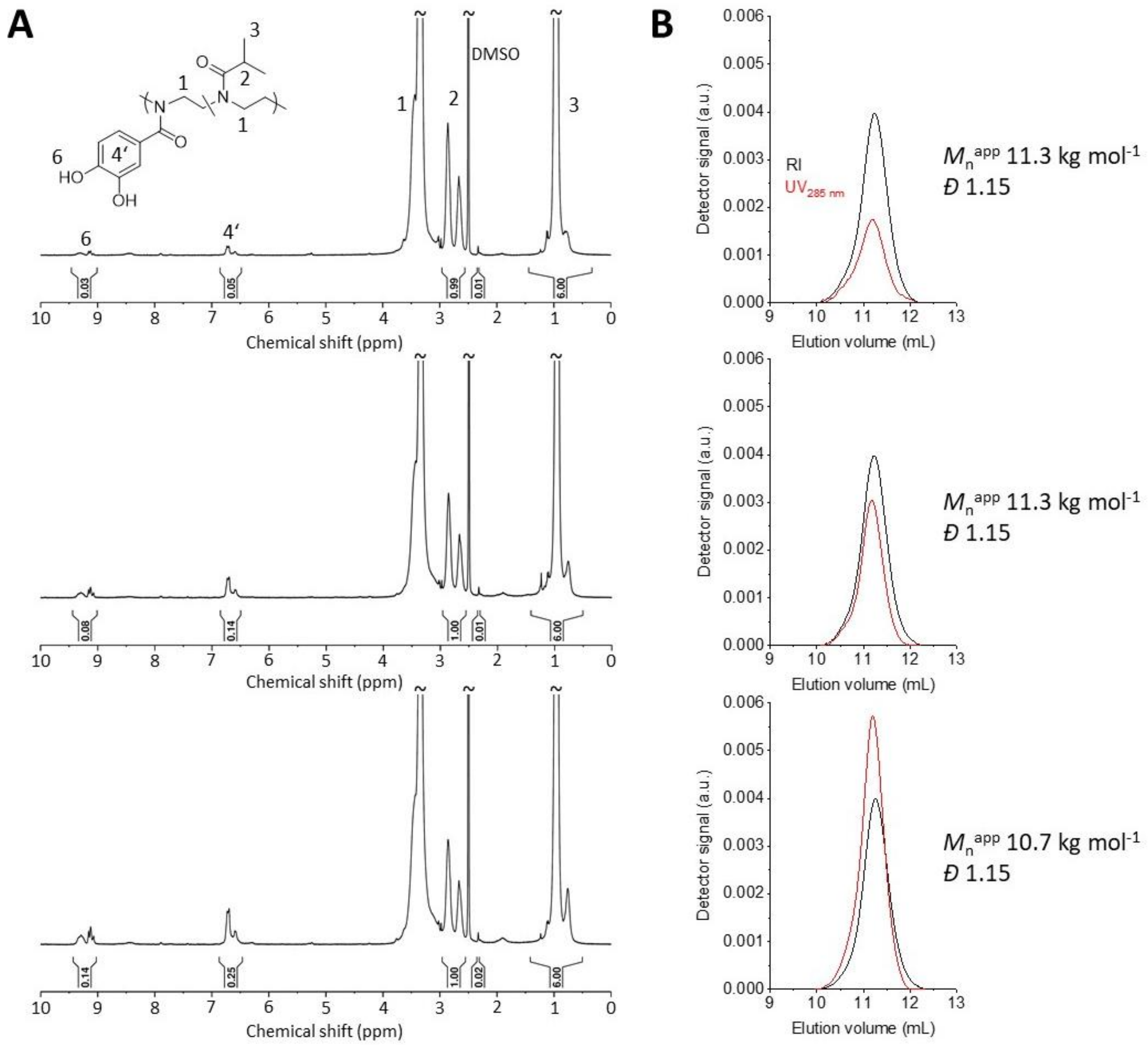

Figure S14. (A) ^{1}H NMR (400 MHz, DMSO-d$_6$) spectra and (B) SEC-RI/UV$_{285\ nm}$ traces (eluent: NMP) of the copolymers $\mathbf{P_{1.9}^{OH}}$, $\mathbf{P_{4.8}^{OH}}$, and $\mathbf{P_{7.9}^{OH}}$ (top to bottom).

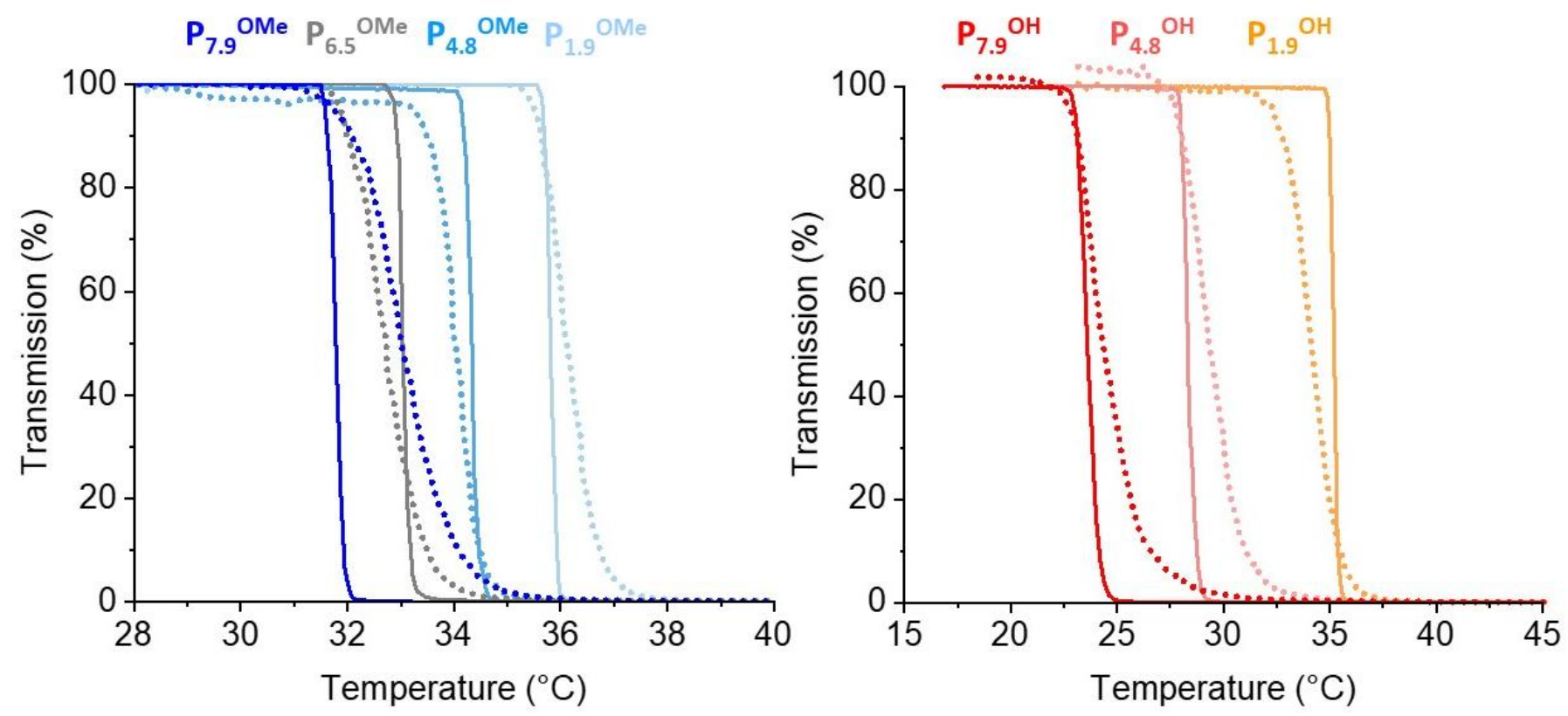

Figure S15. Turbidity curves for 1 wt% solutions of $\mathbf{P_{1.9}^{OMe}}$, $\mathbf{P_{4.8}^{OMe}}$, $\mathbf{P_{6.5}^{OMe}}$ and $\mathbf{P_{7.9}^{OMe}}$ (left) and $\mathbf{P_{1.9}^{OH}}$, $\mathbf{P_{4.8}^{OH}}$, and $\mathbf{P_{7.9}^{OH}}$ (right) in physiological saline solution (154 mM NaCl); heating: solid line, cooling: dotted line, 1 K min^{-1}.

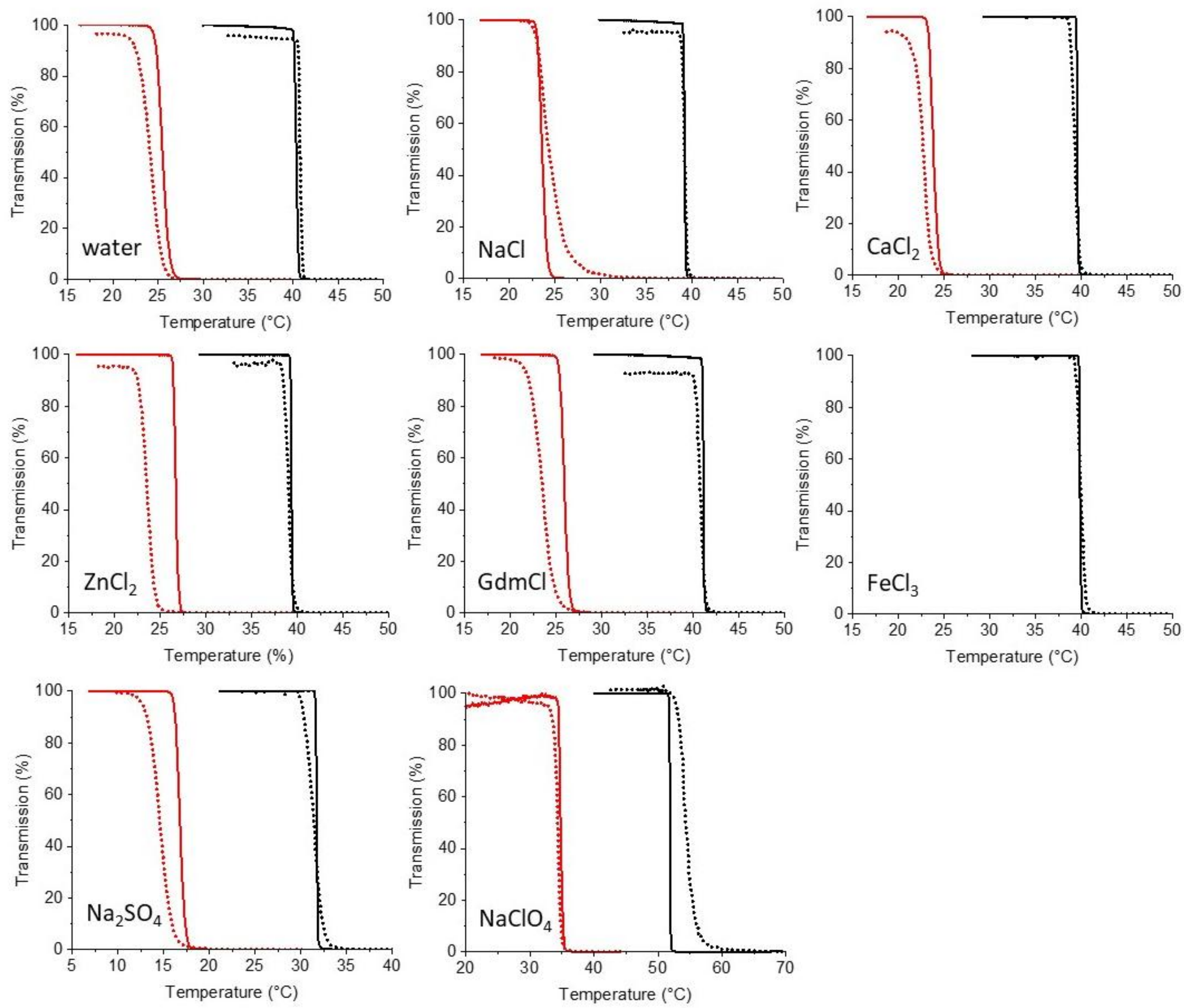

Figure S16. Turbidity curves for 1 wt% solutions of PiPOx (black) and $\mathbf{P_{7.9}^{OH}}$ (red) in water and in 154 mM salt solutions; heating: solid line, cooling: dotted line, 1 K min^{-1}.

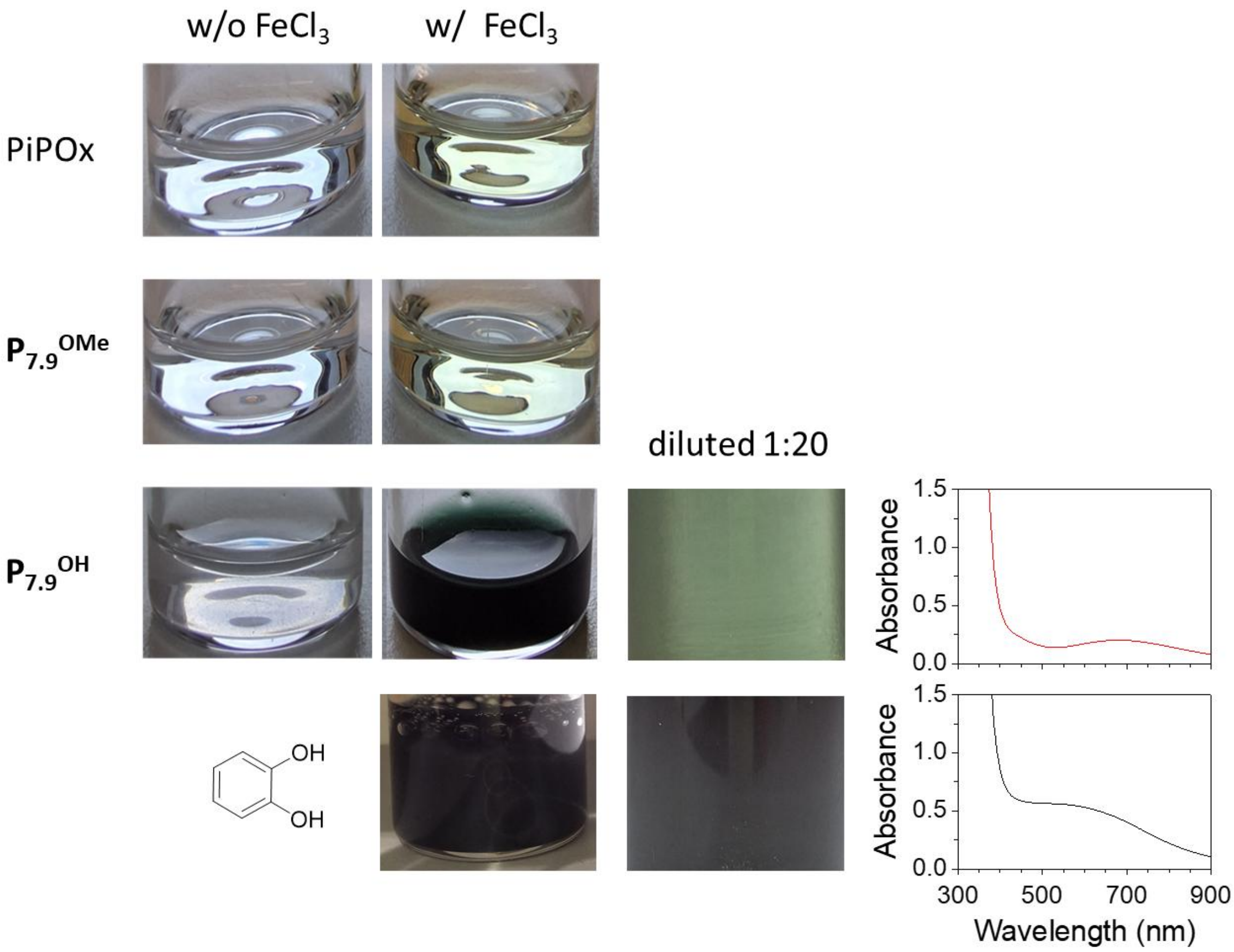

Figure S17. Optical photographs of 1 wt% aqueous solutions of PiPOx, $\mathbf{P_{7.9}}^{\mathbf{OMe}}$, and $\mathbf{P_{7.9}}^{\mathbf{OH}}$ (top to bottom) in the absence and presence of $FeCl_3$ (154 mM) and of a 0.075 wt% solution of 1,2-dihydroxybenzene in 154 mM $FeCl_3$. Right: Optical photographs and UV-visible spectra of 1:20 diluted samples of $\mathbf{P_{7.9}}^{\mathbf{OH}}/Fe^{3+}$ and 1,2-dihydroxybenzene/Fe^{3+}.

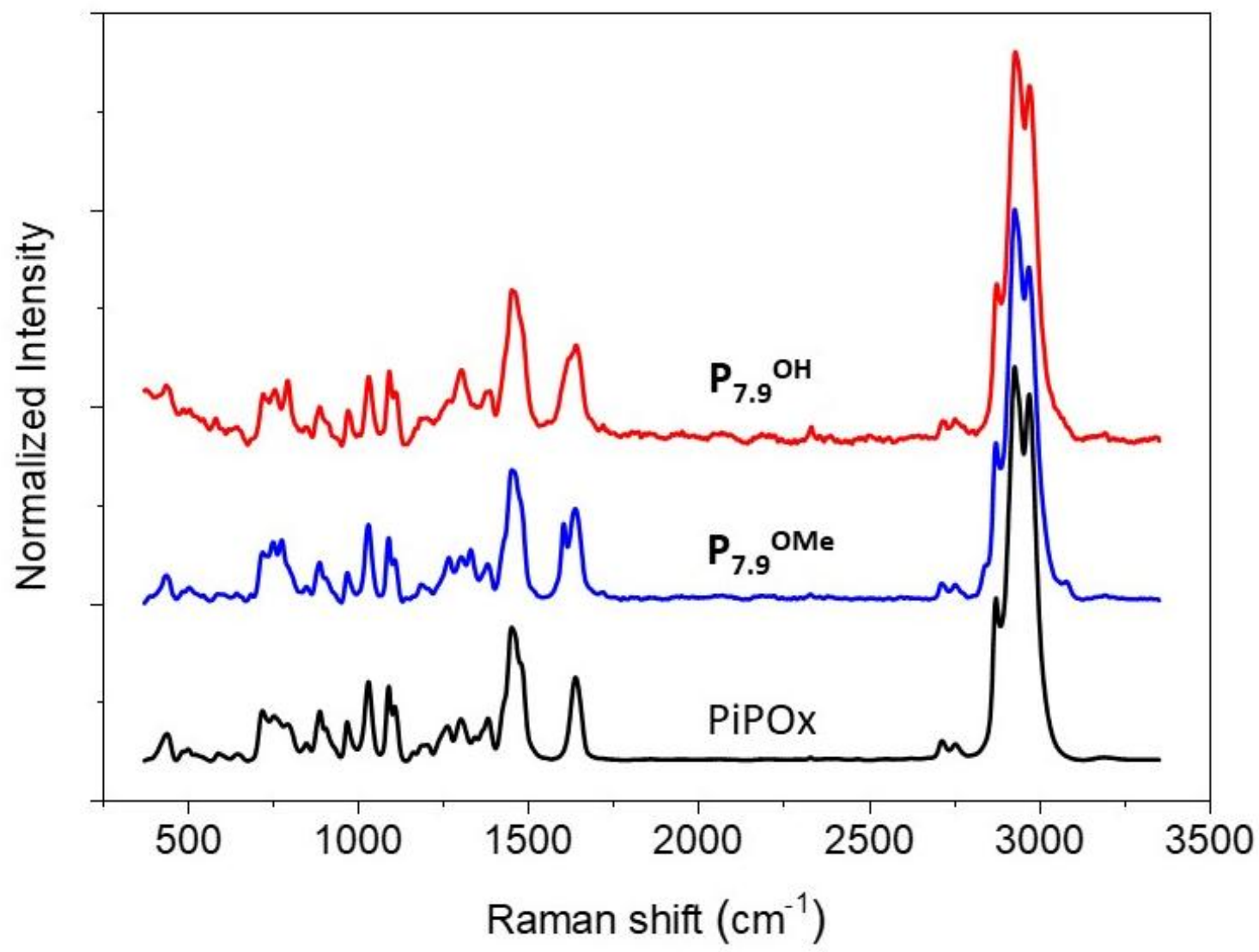

Figure S18. Raman spectra of freeze-dried PiPOx (black), $\mathbf{P_{7.9}^{OMe}}$ (blue), and $\mathbf{P_{7.9}^{OH}}$ (red).

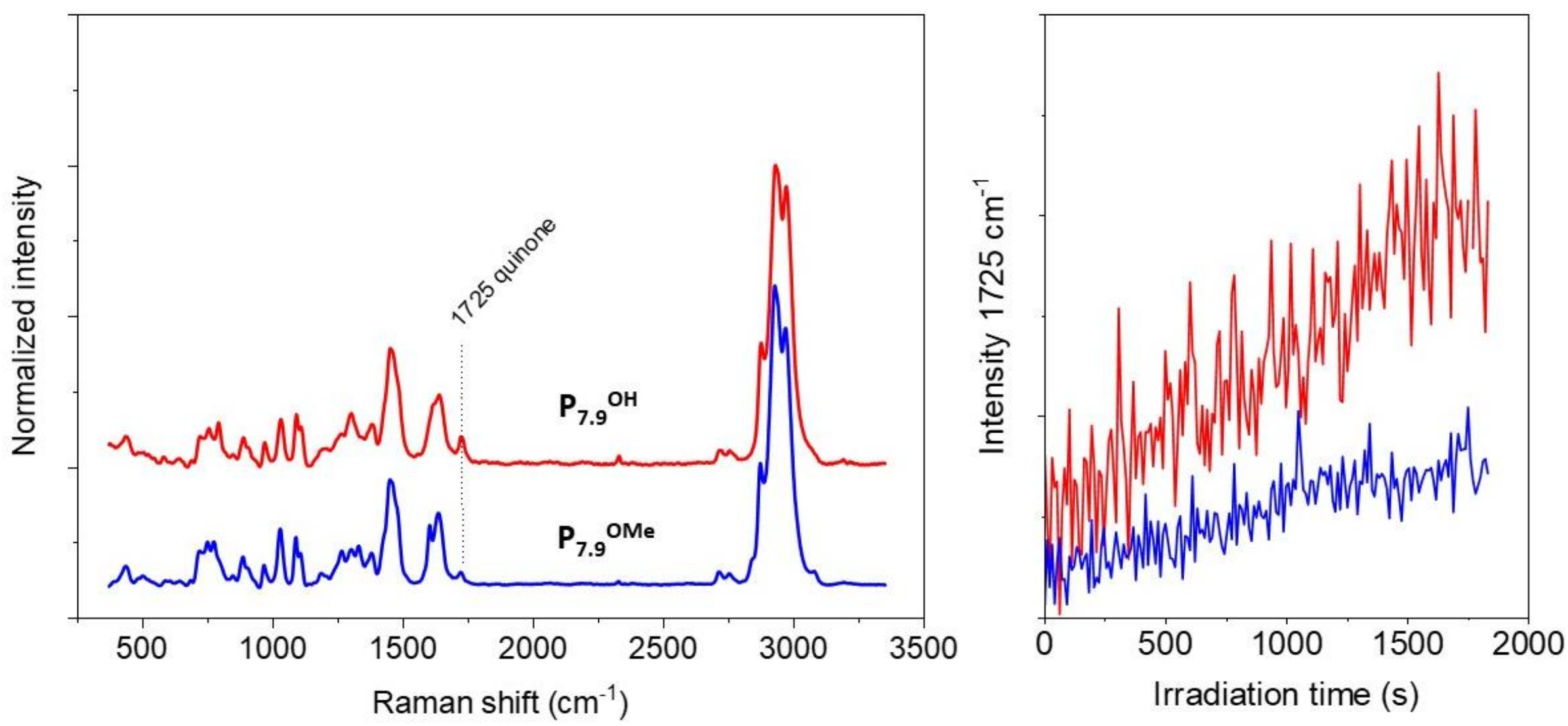

Figure S19. Raman spectra of freeze-dried $\mathbf{P_{7.9}^{OMe}}$ (blue), and $\mathbf{P_{7.9}^{OH}}$ (red) after irradiation with laser light for ~30 min under ambient atmosphere (left) and evolution of the intensity of quinone peak at 1725 cm^{-1} with irradiation time.

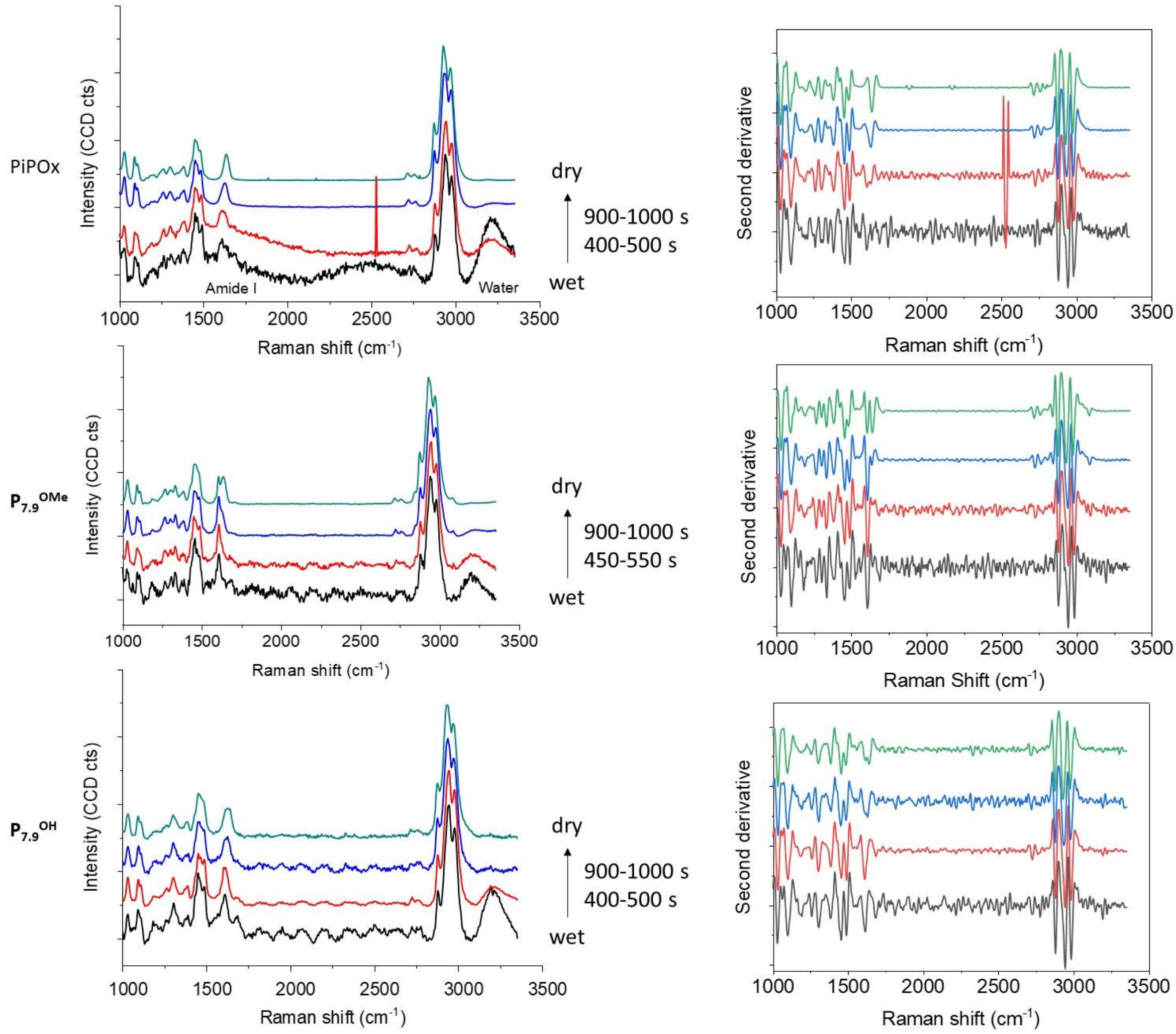

Figure S20. Raman spectra (left) and second derivative (right) of PiPOx, $\mathbf{P_{7.9}^{OMe}}$, and $\mathbf{P_{7.9}^{OH}}$ (top to bottom) obtained during passive dehydration of the samples.
